# PPh_3_ enhanced catalytic conversion of CO_2_ to ethanol over a Ru–Co catalyst

**DOI:** 10.1039/d6sc02731h

**Published:** 2026-08-25

**Authors:** Jia Guo, Ying Wang, Xianyu Meng, Ziyu Li, Shenggui He, Hongxing Wang, Jie Li, Chenglong Yu, Zhuo Zhang, Qingli Qian, Buxing Han

**Affiliations:** a Beijing National Laboratory for Molecular Sciences, CAS Laboratory of Colloid and Interface and Thermodynamics, CAS Research/Education Center for Excellence in Molecular Sciences, Center for Carbon Neutral Chemistry, Institute of Chemistry, Chinese Academy of Sciences Beijing 100190 China qianql@iccas.ac.cn hanbx@iccas.ac.cn; b School of Chemical Science, University of Chinese Academy of Sciences Beijing 100049 China; c College of Chemical Engineering and Materials Science, Tianjin University of Science and Technology Tianjin 300457 China; d Department of Chemical Engineering, The University of Manchester Manchester M13 9PL UK

## Abstract

CO_2_ is a major greenhouse gas as well as an abundant, cheap, and renewable carbon resource. Synthesis of ethanol from CO_2_ using a homogeneous catalyst represents a promising protocol for CO_2_ utilization, yet achieving high catalytic performance with a cost-effective catalyst remains a challenge. Herein, we report a highly efficient Ru–Co–PPh_3_ bimetallic catalyst for ethanol synthesis using CO_2,_ methanol, and H_2_. The ethanol space time yield (STY) reached as high as 4.4 g L^−1^ h^−1^, with a turnover frequency (TOF) of 23.2 h^−1^ and an ethanol selectivity of 85.6 C-mol% in total products, outperforming the previous reports of homogeneous catalytic systems for conversion of CO_2_ to ethanol. The PPh_3_ ligand enabled the high-efficiency reaction by direct utilization of much cheaper Ru–Co precursors and with lower catalyst dosage. Notably, the PPh_3_ ligand played multiple roles in improving the activity, selectivity and stability of the catalyst. The reaction mechanism was also discussed based on a series of control experiments. This work provides a sustainable and practical way of ethanol synthesis using CO_2_ as a feedstock.

## Abstract 2

A multifunctional PPh_3_ ligand enables efficient ethanol synthesis from CO_2_, methanol, and H_2_, using low-cost, readily available and stable Ru–Co halides.

## Graphical abstract

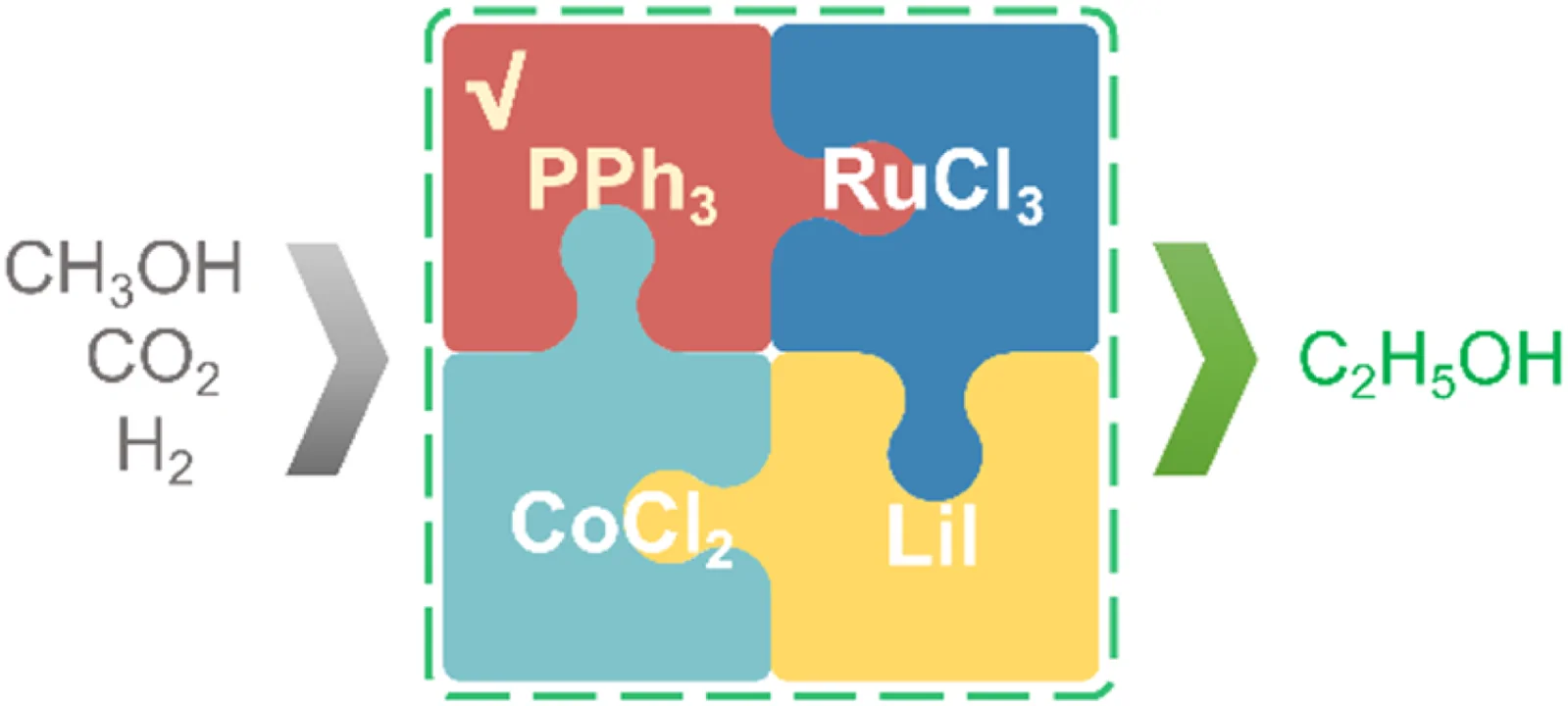

## Introduction

Carbon dioxide (CO_2_), as a major greenhouse gas as well as a renewable, non-toxic, and abundant C_1_ resource, holds significant importance for environmental protection through its conversion into value-added products.^1–7^ CO_2_ can be hydrogenated to synthesize different chemicals, such as methanol,^8–12^ ethanol,^13^ acetamides,^14^ liquid fuel,^15–20^ formic acid,^21–24^ acetic acid^25^ and higher carboxylic acids.^26–28^ Among these, methanol is a versatile chemical feedstock, and its production from CO_2_ has been put into industrial application.^7,8^ As a high-value C_2_ oxygenate, ethanol serves as both an essential bulk chemical and a clean fuel additive with higher energy density, lower toxicity and broader applicability.^29^ Therefore, the efficient synthesis of ethanol from CO_2_ exhibits a promising route for carbon neutrality. Nevertheless, this reaction is more challenging because it involves controllable CO_2_ hydrogenation and simultaneous C–C bond formation.

Recent advances in the thermal catalytic hydrogenation of CO_2_ to ethanol have focused primarily on the design of heterogeneous catalysts including different transition metals.^30–38^ Nevertheless, heterogeneous catalysts face challenges such as higher reaction temperatures and complex preparation processes. In contrast, homogeneous catalytic systems have shown promising potential for ethanol production under milder conditions, such as those based on Ru,^39^ Ru–Co^40–45^ and Ru–Rh^46^ catalysts in different solvents. A general reaction mechanism for CO_2_ hydrogenation to ethanol with homogeneous Ru–Co catalytic systems has been proposed: CO_2_ is first hydrogenated to CO *via* the reverse water–gas shift (RWGS) reaction over the Ru catalyst. The *in situ* generated CO subsequently serves as the key C_1_ intermediate, undergoing C–C coupling with 
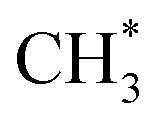
 derived from methanol, anisole, formaldehyde, or dimethyl ether in the presence of the Co catalyst. Then acetaldehyde is generated by reductive elimination, which is *in situ* hydrogenated to ethanol. However, the existing homogeneous catalytic systems still commonly suffer from drawbacks such as expensive catalyst precursors (Table S1), and their catalytic efficiencies are to be further enhanced for possible practical application.

Herein, we report the efficient synthesis of ethanol from CO_2_, methanol, and H_2_ using the Ru–Co–PPh_3_ catalytic system (Fig. 1). Currently, CO_2_-to-methanol is already industrialized, and our work bridges it to higher-value chemicals, advancing sustainable carbon utilization. The PPh_3_ ligand enabled the reaction by direct utilization of much cheaper and easily available RuCl_3_·3H_2_O and CoCl_2_·6H_2_O as catalyst precursors. The catalytic system demonstrated a remarkably higher production rate and selectivity of ethanol compared with those in previous reports (Table S1). Under optimized conditions, our catalytic system (entry 6) achieved significantly higher catalytic activity (STY = 4.4 g L^−1^ h^−1^, TOF = 23.2 h^−1^) and superior ethanol selectivity (85.6 C-mol%) compared to previously reported homogeneous systems (entries 1–5, STY = 1.7–3.4 g L^−1^ h^−1^, TOF = 2.5–17.9 h^−1^, *S*_ethanol_ = 50.3–71.7 C-mol%). In addition, this superior performance is accomplished with cost-effective catalyst precursors, lower catalyst dosages, and a shorter reaction time (5 h). Specifically, the cost of our Ru precursor (RuCl_3_·3H_2_O) is 13–72 times lower, and that of our Co precursor (CoCl_2_·6H_2_O) is 3–194 times lower than that of the corresponding precursors used in previous systems (entries 1–5). Interestingly, the PPh_3_ ligand played multiple roles in enhancing the activity, selectivity and stability of the catalyst. This work provides an economical, efficient, and easily operable route for CO_2_ utilization and the green synthesis of ethanol.

**Fig. 1:**
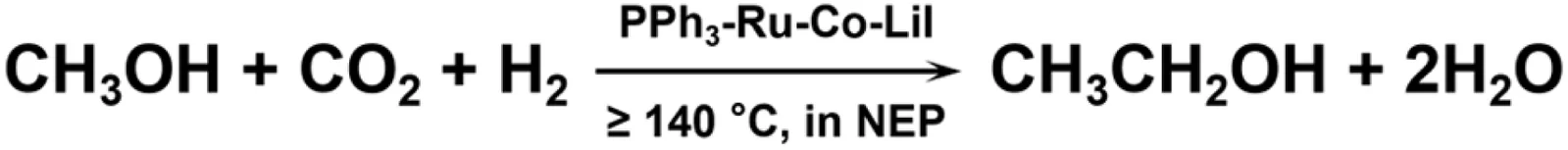
Synthesis of ethanol by methanol homologation with CO_2_ and H_2_.

## Catalytic results

### Catalytic system

To explore an economic, stable and efficient catalytic system for ethanol synthesis, various catalytic systems were evaluated, as shown in Table 1. All the reactions were performed in a 16 mL Teflon-lined stainless-steel autoclave with a magnetic stir bar (Fig. S1–S3). Considering experimental safety, we screened the catalytic system and basic reaction conditions at a relatively lower H_2_ pressure of 6.5 MPa. The RuCl_3_·3H_2_O/CoCl_2_·6H_2_O bimetallic catalysts, in combination with PPh_3_ as the ligand and LiI as the promoter in 1-ethyl-2-pyrrolidone (NEP) solvent, could efficiently promote the reaction, where an ethanol STY of 3.5 g L^−1^ h^−1^ and ethanol selectivity of 72.4 C-mol% were obtained (entry 1). The CO_2_ conversion rate was determined to be 17.2% in the reaction, and the turnover frequency (TOF) of the catalyst for ethanol production was 18.3 h^−1^. The representative graphs of GC and GC-MS are shown in Fig. S4 and S5. We also investigated the change in actual total pressure during the reaction process (Fig. S6). The results showed that the total pressure first increased from 8.0 MPa to about 10.7 MPa with increasing temperature, and then gradually decreased to about 10.4 MPa at the end of the reaction (5 h), corresponding to the consumption of gaseous reactants (CO, CO_2_ and H_2_) during the reaction. The space-time yield (STY) directly correlates with the production capacity of an industrial reactor, making it a critical metric for evaluating homogeneous catalysts. Given the strong industrial application potential of our catalyst, we adopted STY as the primary metric to assess ethanol production rates.

**Table 1:** Different catalytic systems for ethanol synthesis from methanol, CO_2_ and H_2_^a^

Entry	Catalyst precursors	Ligand	Promoter	STY of ethanol^b^	Selectivity (C-mol%)		
					Ethanol	CH_4_	Methyl acetate
1^c^	RuCl_3_·3H_2_O, CoCl_2_·6H_2_O	PPh_3_	LiI	3.5	72.4	27.6	0
2	RuCl_3_·3H_2_O, CoCl_2_·6H_2_O	—	—	0	0	100	0
3	RuCl_3_·3H_2_O, CoCl_2_·6H_2_O	—	LiI	2.4	52.4	46.7	0.9
4	RuCl_3_·3H_2_O, CoCl_2_·6H_2_O	PPh_3_	—	0	0	100	0
5	RuCl_3_·3H_2_O, CoCl_2_·6H_2_O	PPh_3_	NaI	3.4	68.0	32.0	0
6	RuCl_3_·3H_2_O, CoCl_2_·6H_2_O	PPh_3_	KI	3.4	63.1	36.9	0
7	RuCl_3_·3H_2_O, CoCl_2_·6H_2_O	PPh_3_	LiBr	0.7	42.8	57.2	0
8	RuCl_3_·3H_2_O, CoCl_2_·6H_2_O	PPh_3_	LiCl	0.2	52.2	47.8	0
9	RuCl_3_·3H_2_O	PPh_3_	LiI	0.2	12.6	86.4	1.0
10	CoCl_2_·6H_2_O	PPh_3_	LiI	0	0	0	0
11	RuCl_3_·3H_2_O, Co(BF_4_)_2_·6H_2_O	PPh_3_	LiI	1.2	26.0	51.3	22.7
12	RuCl_3_·3H_2_O, CoBr_2_	PPh_3_	LiI	3.1	61.5	38.5	0
13	RuI_3_, CoCl_2_·6H_2_O	PPh_3_	LiI	3.4	59.7	40.3	0
14	Ru(PPh_3_)_3_Cl_2_, Co(PPh_3_)_3_Cl	PPh_3_	LiI	1.5	71.2	27.8	1.0
15^d^	Ru_3_(CO)_12_, Co_4_(CO)_12_	PPh_3_	LiI	2.8	74.5	24.6	0.9
16	Ru(bpy)_3_Cl_2_·6H_2_O, Co_2_(CO)_8_	PPh_3_	LiI	0	0	100	0

a Reaction conditions: 10 µmol Ru catalyst and 45 µmol Co catalyst (based on the metal), 2.3 mmol promoter, 40 µmol ligand, 2 mL NEP, 10 mmol methanol, 0.2 MPa CO, 1.3 MPa CO_2_ and 6.5 MPa H_2_ (at room temperature), 180 °C, 5 h.

b STY stands for space time yield of ethanol (g L^−1^ h^−1^). The STY was determined by GC analysis using toluene as the internal standard.

c Yield of ethanol based on the methanol feedstock was 8.8% and TOF of ethanol was 18.3 h^−1^ (TOF denotes moles of ethanol produced per mole of the Ru catalyst per hour).

d A black precipitate was observed after the reaction, indicating the decomposition of metal complexes and formation of fine metal powder.

Based on prior research confirming CO as an intermediate in converting CO_2_ to ethanol,^41,42^ and to better align with continuous industrial conditions, we carried out catalyst screening by introducing a small amount of CO (1.3 mmol) into the reactor. LiI is an indispensable promoter of the reaction (entries 1–4). Further investigation revealed that other iodides (NaI and KI) may also promote ethanol generation with similar STY values (entries 5 and 6), and the contribution of the iodide promoters to ethanol selectivity decreased in the order LiI > NaI > KI. When we used other lithium halides (LiCl and LiBr) instead of the LiI promoter, the STY and selectivity of ethanol were very low, which suggested that the iodide anion was indispensable (entries 7 and 8). Notably, the PPh_3_ ligand played a critical role in modulating the catalytic performance. A direct comparison between entry 1 (with PPh_3_) and entry 3 (without ligand) revealed that PPh_3_ significantly boosted both STY (from 2.4 to 3.5 g L^−1^ h^−1^) and ethanol selectivity (from 52.4 to 72.4 C-mol%), while drastically suppressing CH_4_ formation (from 46.7 to 27.6 C-mol%).

When RuCl_3_·3H_2_O was utilized alone as the catalyst, both the STY and selectivity of ethanol decreased remarkably (entry 9), while no ethanol was generated with CoCl_2_·6H_2_O as the single catalyst to catalyze the reaction (entry 10). These results showed that Ru is the major catalyst and a remarkable synergistic effect existed between the Ru and Co catalysts in promoting the desired reaction. We also conducted experiments with other Ru–Co combinations (entries 11–16). In the presence of the PPh_3_ ligand, the RuCl_3_·3H_2_O and CoCl_2_·6H_2_O pair demonstrated the best balance of STY, selectivity and catalyst stability, which are critical advantages over other more expensive or unstable alternatives.

### Ligand effects

To further investigate ligand effects, we examined a series of ligands (Fig. 2). First, we screened the ligands with different atoms, like P, N and B. The results showed that ligands containing P or N atoms could facilitate ethanol synthesis, whereas the ligand bearing B atom inhibited the desired reaction. This may be attributed to the stronger σ-donor ability and appropriate π-acceptor properties of P/N donors, which could stabilize the catalytic system and facilitate the formation of ethanol.

**Fig. 2:**
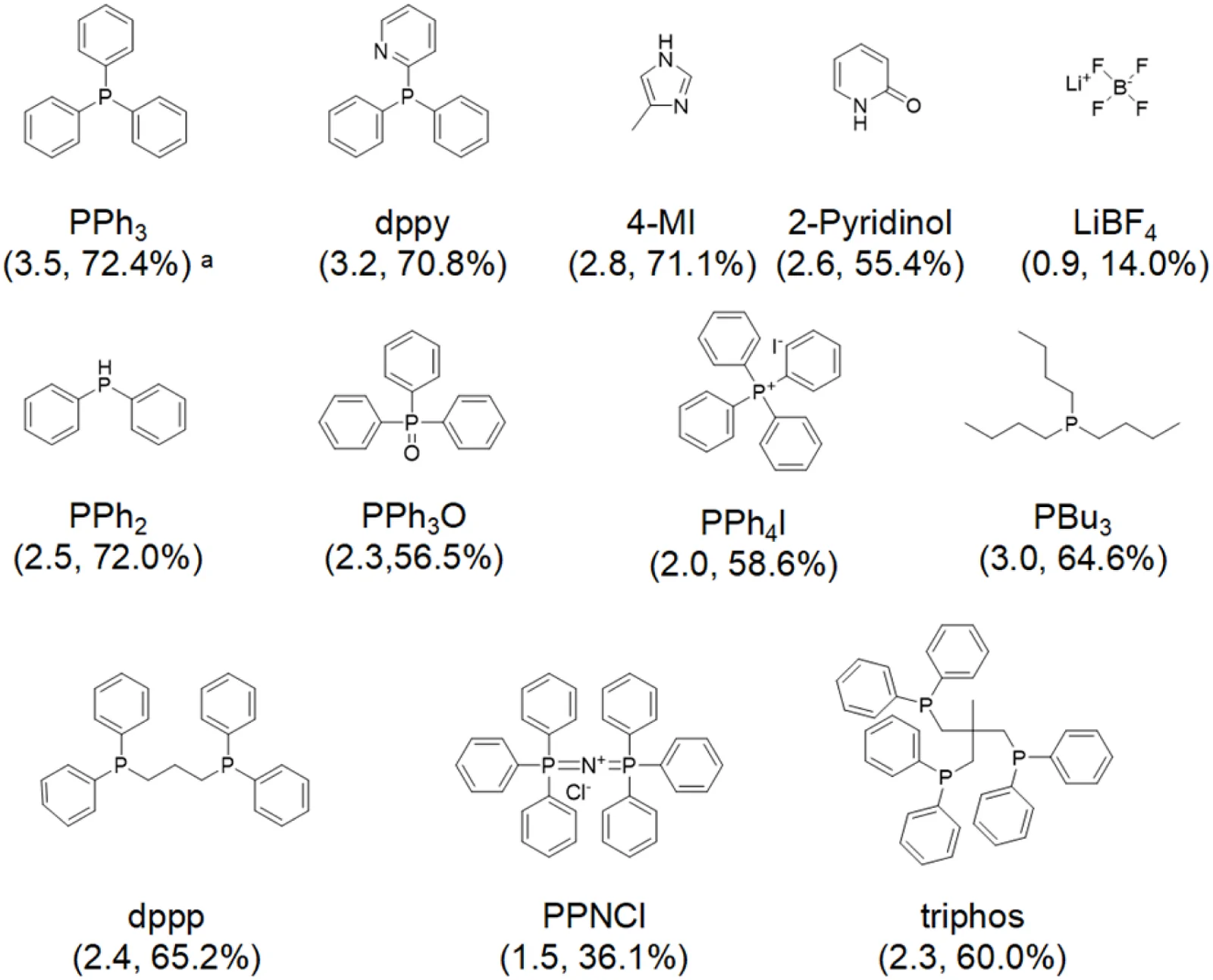
Effects of different ligands on STY and selectivity of ethanol synthesis. 40 µmol ligand (based on the coordinating atom) was used, and other conditions were the same as those in entry 1 in Table 1. ^*a*^(3.5, 72.4%) means the STY of ethanol is 3.5 g L^−1^ h^−1^, and the selectivity of ethanol is 72.4 C-mol%. Abbreviations: PPh_3_ (triphenylphosphine), dppy (diphenyl-2-pyridylphosphine), 4-MI (4-methylimidazole), 2-pyridinol (2-hydroxypyridine), PPh_2_ (diphenylphosphine), PPh_3_O (triphenylphosphine oxide), P(Ph)_4_I (tetraphenylphosphonium iodide), PBu_3_ (tributylphosphine), dppp (1,3-bis(diphenylphosphino)propane), PPNCl (bis(triphenylphosphine)iminium chloride), and triphos (1,1,1-tris(diphenylphosphinomethyl)ethane).

Phosphine ligands, in particular, were found to play a significant role in modulating catalytic performance. Further studies on substituent variations of the phosphine ligand revealed that PPh_2_ and P(Ph)_4_I led to a remarkable decrease in catalytic performance, which indicated that the number of phenyl groups on the phosphine center could strongly influence the target reaction. In addition, triphenylphosphine oxide was found to inhibit the synthesis of ethanol. Replacing the phenyl groups on the phosphine atom with butyl groups led to inferior catalytic performance, confirming the superiority of the phenyl-substituted phosphine ligand for ethanol synthesis.

We also examined the effect of the different denticities of the phosphine ligand. Notably, the catalytic activity decreased with the increasing denticity of the phosphine ligand. Specifically, bidentate (dppp) and tridentate phosphine ligands (triphos) were less effective than the monodentate phosphine ligand (PPh_3_), and the ligand effect followed the order PPh_3_ > dppp > triphos. This trend may be explained by the smaller steric hindrance of PPh_3_, which provided more accessible coordination sites. In brief, it was found that PPh_3_ remained the appropriate ligand, which achieved the highest activity and selectivity toward ethanol formation.

### Solvent effects

The solvent effect was crucial for the reaction and a systematic investigation was conducted (Fig. S7). The solvent containing cyclic amide structure with an *N*-alkyl substituent was found to promote ethanol synthesis by methanol homologation using CO_2_.^42^ Please see the SI for more detailed discussion. NEP proved to be the most effective solvent for promoting ethanol synthesis. Besides the molecular structure of the solvent, the peculiar high-temperature solubility and thermodynamic correlation of gaseous reactants and byproducts (CO_2_, H_2_, and CH_4_) in NEP also help to understand its role in the catalytic reaction.^47^

The effect of the volume ratio of methanol to NEP on catalytic performance was systematically investigated (Fig. 3), revealing a clear volcano-type trend in both ethanol space-time yield (STY) and selectivity. In the absence of methanol (0.0/2.0), the target reaction was nearly stagnant: ethanol STY was negligible and selectivity was below 20 C-mol%, confirming that methanol acts as an indispensable carbon substrate for ethanol formation. As the methanol/NEP ratio increased from 0.05/2.0 to 0.4/2.0, the ethanol STY increased sharply from ∼1.2 to 3.5 g L^−1^ h^−1^, while ethanol selectivity climbed rapidly to a plateau of ∼70–73 C-mol%. This enhancement can be attributed to the increased availability of the methanol substrate, which drives the carbon chain growth toward ethanol. When the methanol/NEP ratio was further increased, a trend of decrease in STY and selectivity of ethanol began. A considerable drop in both STY and selectivity was observed at 2.0/0.0 (pure methanol), destabilizing the catalyst and favouring side reactions. These results highlighted the critical role of the methanol/NEP ratio in balancing reaction activity, selectivity, and catalyst stability, and reaction with 0.4 mL methanol (10 mmol) and 2.0 mL NEP was the suitable solvent condition for efficient ethanol synthesis.

**Fig. 3:**
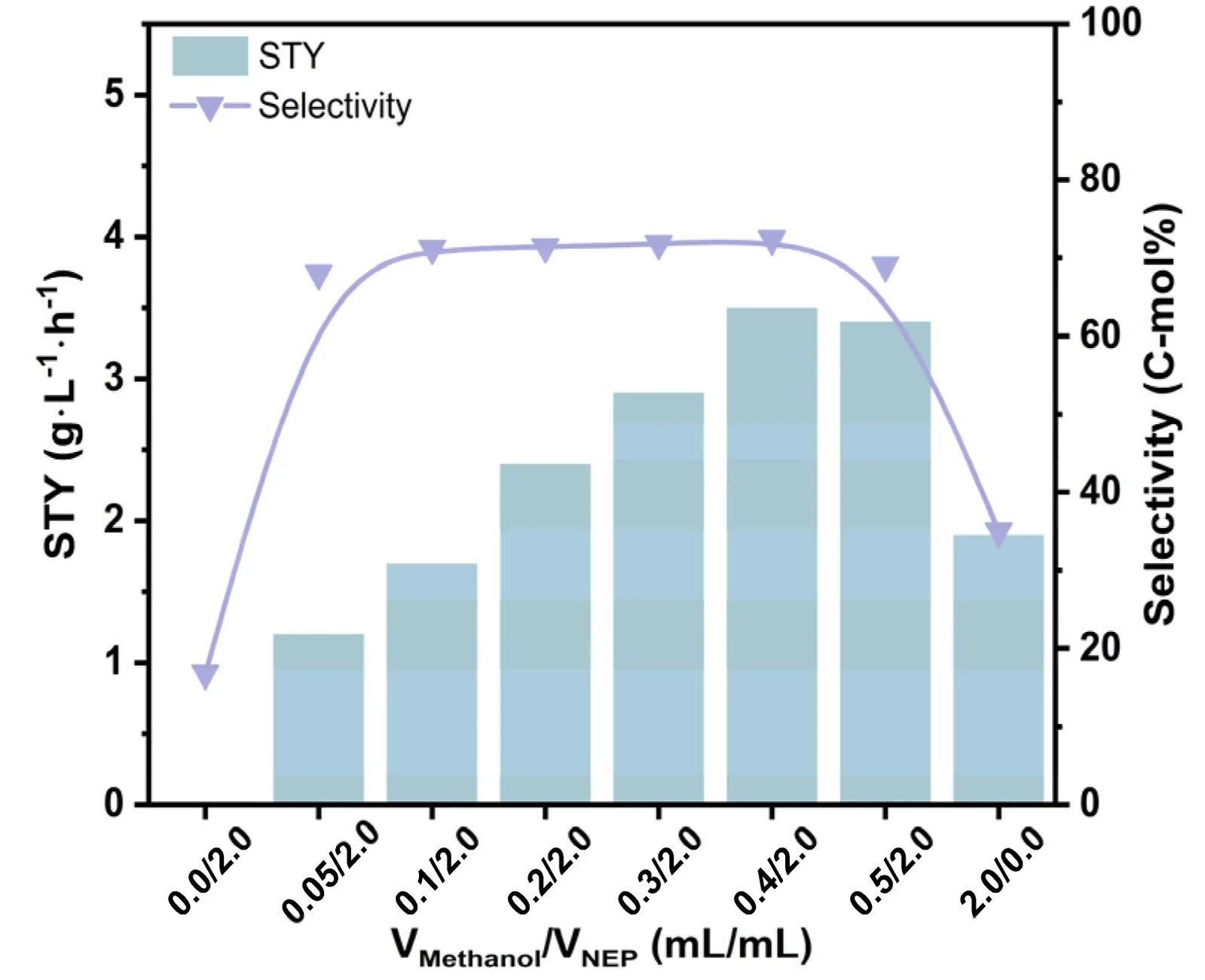
The impact of the methanol/NEP ratio on the reaction results. Other reaction conditions were the same as those in entry 1 in Table 1.

### Effects of reaction parameters

The impact of reaction temperature is shown in Fig. 4. Ethanol began to emerge at 140 °C, and the STY and selectivity of ethanol increased evidently with increasing temperature. Better reaction performance was obtained at 180 °C (an ethanol STY of 3.5 g L^−1^ h^−1^ and ethanol selectivity of 72.4 C-mol%). However, the ethanol selectivity decreased obviously when the temperature was further elevated, which may be attributed to the marked formation of the methane byproduct. Thus, 180 °C was the appropriate reaction temperature for this reaction.

**Fig. 4:**
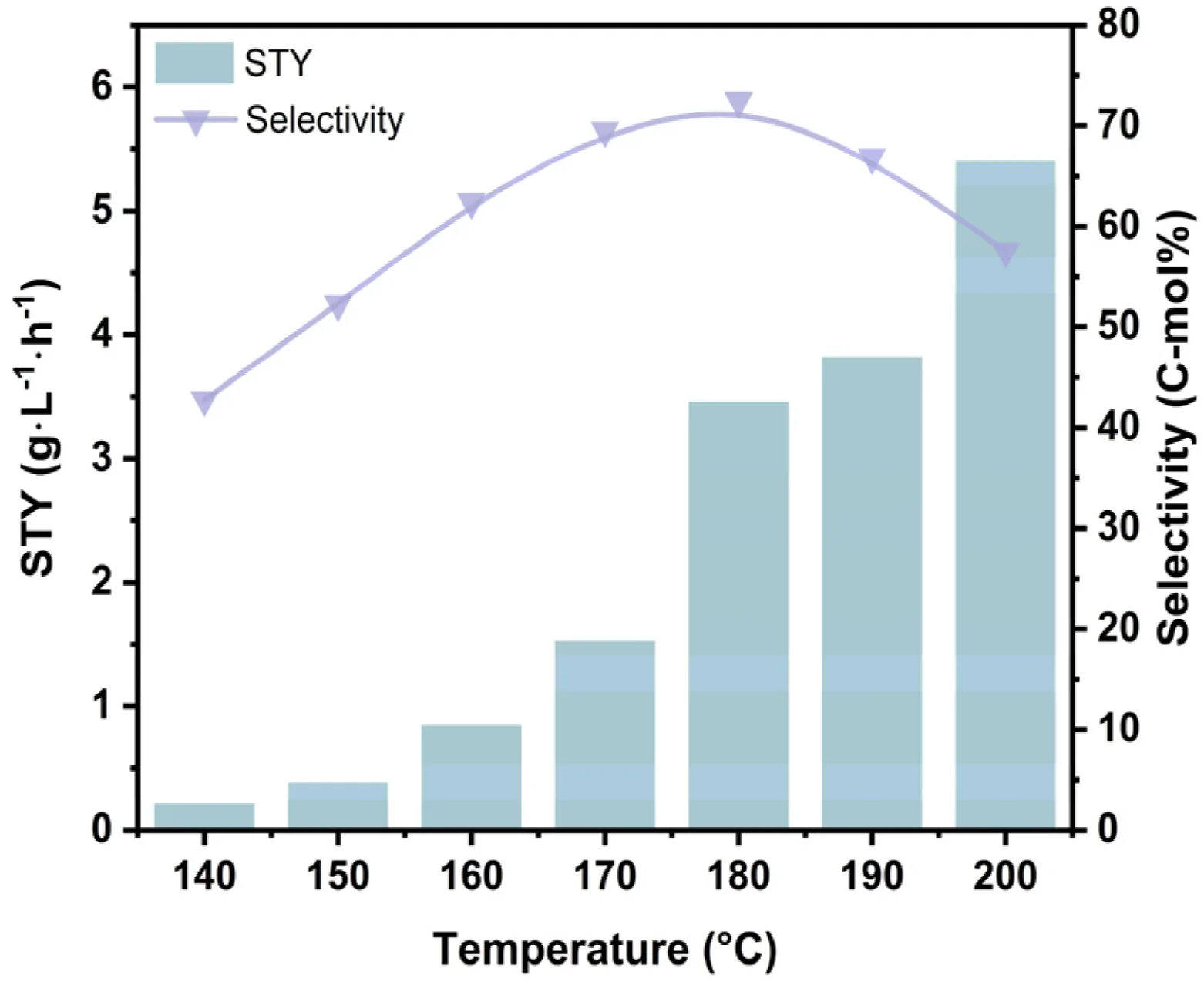
The STY and selectivity of ethanol at different temperatures. Other reaction conditions were the same as those in entry 1 in Table 1.

The apparent activation energy (*E*_a_) for ethanol formation was estimated from the Arrhenius plot using the data in the temperature range from 413 to 453 K (Fig. 5 and Table S2), and the apparent activation energy of this reaction was 110 kJ mol^−1^. At 180 °C, we further investigated the influence of other reaction parameters, including dosages of the transition metal precursors and gas pressures (Table S3). The dosages of Ru and Co were found to affect the target reaction significantly. At the same total amount of metals (55 µmol), the appropriate molar ratio of Ru/Co catalysts was 2/9. At this Ru/Co ratio, when the total amount of the catalysts was changed, 10 µmol Ru and 45 µmol Co gave the best catalytic result. The impact of the dosages of the LiI promoter and PPh_3_ ligand was also studied (Fig. 6). The dosage of LiI affected the reaction activity and selectivity, and 2.3 mmol LiI resulted in the best performance (Fig. 6a). It was indicted that too much I^−^ derived from LiI may occupy the active sites of the catalyst, thereby inhibiting the catalytic reaction. The STY and selectivity of ethanol improved gradually as the dosage of PPh_3_ was increased from 0 µmol to 40 µmol (Fig. 6b). However, further increasing the amount of PPh_3_ to 80 µmol led to a notable decrease in ethanol STY, which can be interpreted by the occupation of the active sites by the excess PPh_3_. Hence, the appropriate dosage of PPh_3_ was 40 µmol when the pressure of H_2_ was 6.5 MPa.

**Fig. 5:**
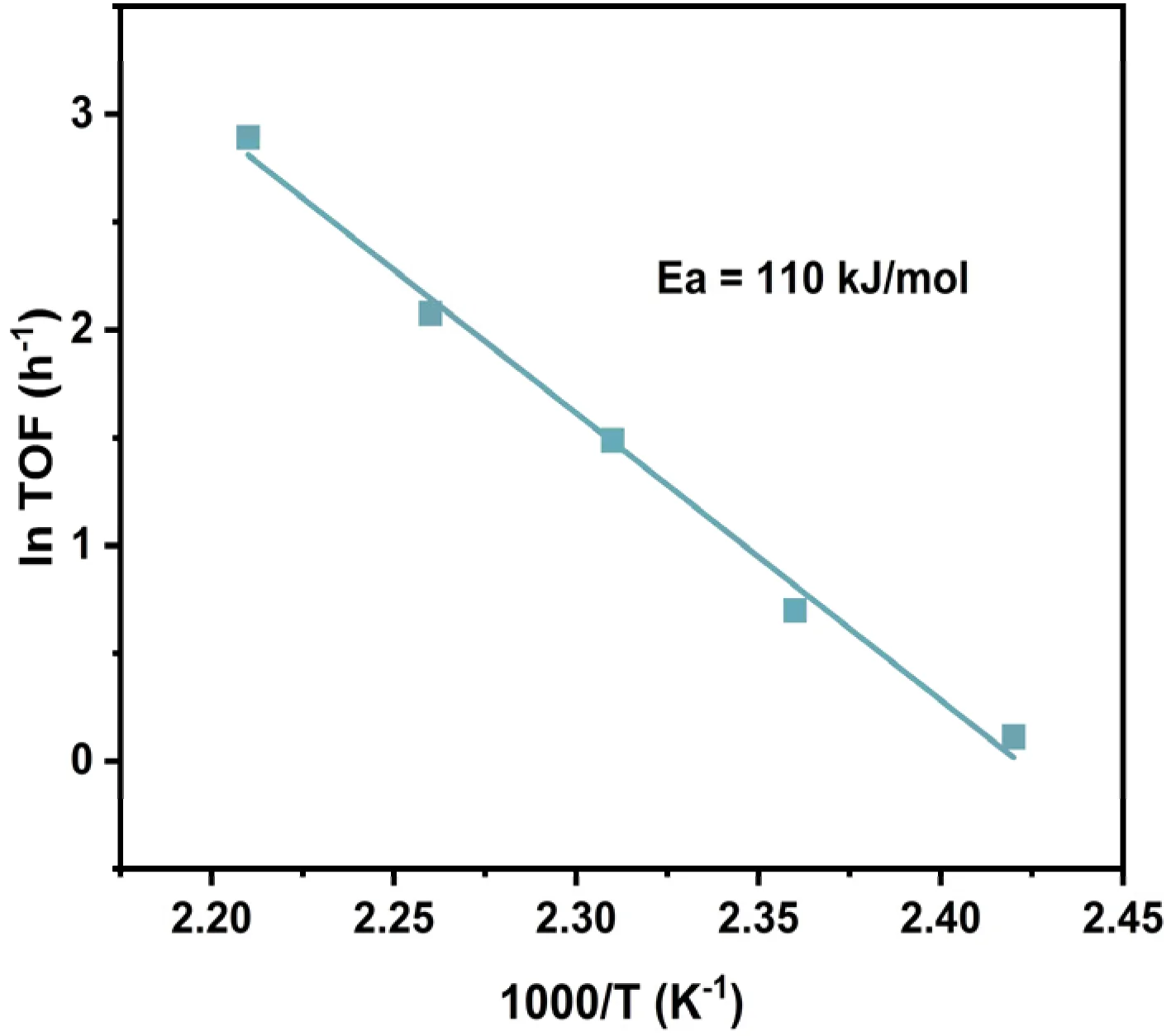
Arrhenius plots for ln TOF (h^−1^) *versus* the inversed temperature (from 413 to 453 K). Other reaction conditions were the same as those in entry 1 in Table 1.

**Fig. 6:**
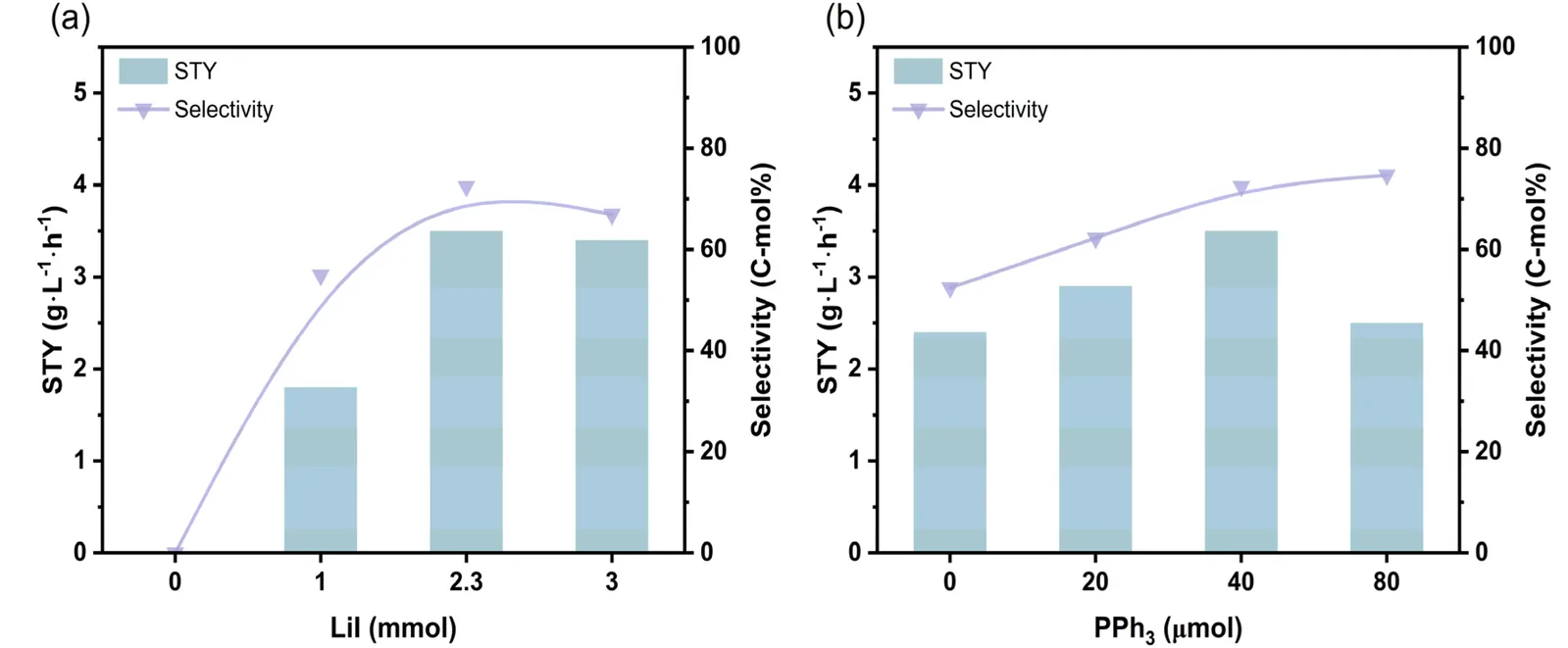
Effects of LiI and PPh_3_ dosages on ethanol synthesis. (a) Effect of LiI dosage; (b) effect of PPh_3_ dosage. Reaction conditions: 2.3 mmol LiI or 40 µmol PPh_3_; other conditions were the same as those in entry 1 in Table 1.

The coexistence of the reactant gases (CO_2_/CO/H_2_) help to enhance the overall catalytic performance, and the effects of the partial pressures of CO_2_, CO, and H_2_ were investigated (Table S3). The results showed that the STY and selectivity of ethanol reached 4.1 g L^−1^ h^−1^ and 76.0 C-mol% in total products when the pressure of CO/CO_2_/H_2_ was 0.2/1.3/8.5 MPa. Notably, a large amount of methyl acetate (by-product) was generated with the increasing proportion of CO, which suggested a synergistic conversion of CO_2_ and CO. Interestingly, the STY and selectivity of ethanol were boosted by the increase in H_2_ pressure until 8.5 MPa H_2_. However, further elevation of H_2_ pressure failed to improve the catalytic performance. We subsequently optimized other reaction conditions under 8.5 MPa H_2_, and better catalytic performance was achieved with an ethanol STY of 4.4 g L^−1^ h^−1^ and an ethanol selectivity of 85.6 C-mol% (Table S4). We would like to mention that the overall performance of this catalytic system is remarkably higher than those reported in the previous reports (Table S1).

### Time course of the reaction

Fig. 7a illustrates the contents of reactants and products during the time course of the reaction. The amounts of methanol and CO_2_ decreased continuously as the reaction proceeded. Ethanol was first detected at 1 h, and its yield increased progressively over time. During the whole course, methane formation increased slowly and steadily. The content of CO decreased gradually in the initial stage (0–3 h), indicating the small amount of CO charged before the reaction was consumed. Subsequently, the CO content stabilized at a constant level with the continuous growth of ethanol production, confirming the *in situ* generation of more CO by the reverse water gas shift (RWGS) reaction. It is known that CO is an intermediate for ethanol formation from methanol, CO_2_ and H_2_. The influence of reaction time on STY and selectivity of ethanol is revealed in Fig. 7b. The ethanol STY increased from ∼1.3 g L^−1^ h^−1^ at 1 h to a maximum of ∼3.5 g L^−1^ h^−1^ at 5 h, and then gradually declined to ∼2.7 g L^−1^ h^−1^ at 9 h. The ethanol selectivity followed a similar trend and the highest selectivity of 72.4 C-mol% was also obtained at 5 h.

**Fig. 7:**
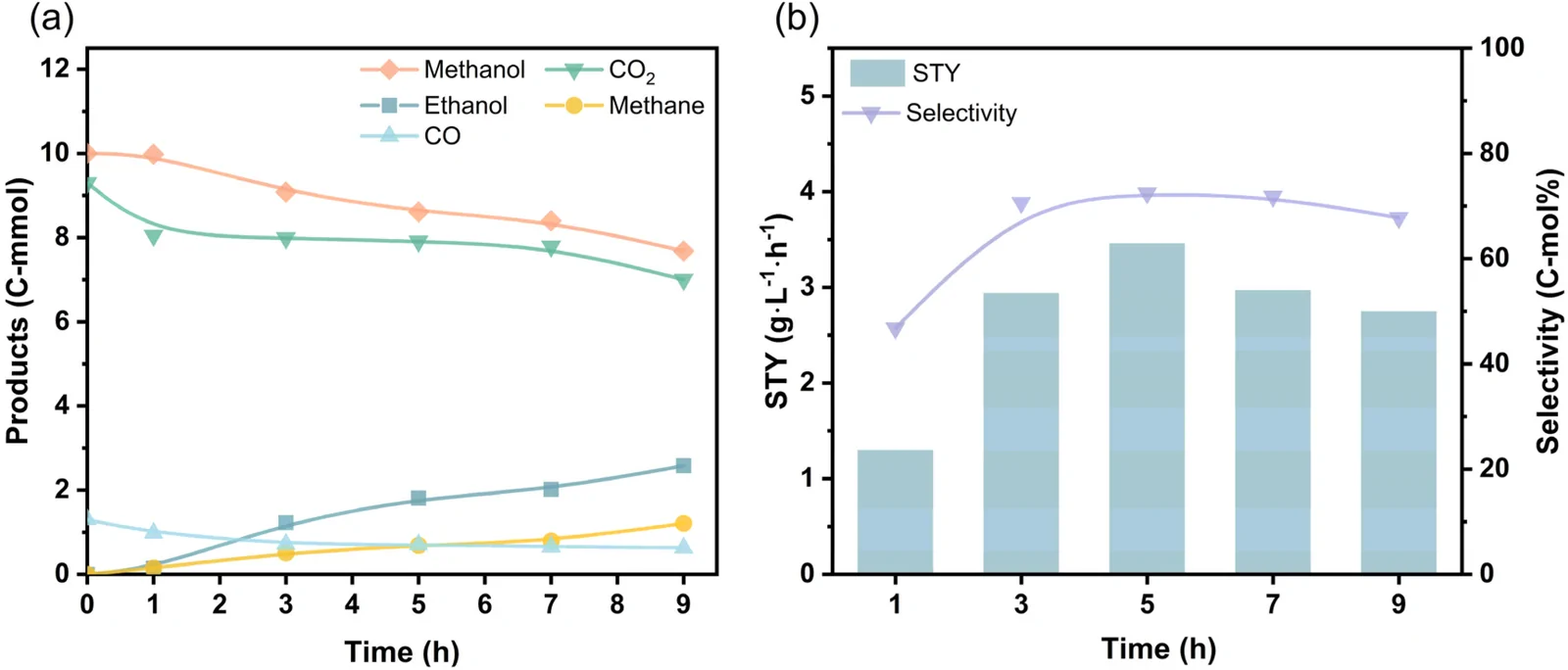
Time course of the reaction. (a) Reactant and product profiles; (b) STY and selectivity of ethanol. Other reaction conditions were the same as those in entry 1 in Table 1.

### Recyclability of the catalytic system

The recyclability of the catalytic system was evaluated. After the reaction, the gases were released after cooling the reactor, and the gaseous and liquid samples were determined by gas chromatography. Ethanol and unreacted methanol were then removed from the reactor under vacuum, and the catalytic system was reused directly for the next reaction. Fig. 8 reveals that the STY and selectivity of ethanol did not decrease at all after five runs, demonstrating the excellent stability and reusability of the catalytic system.

**Fig. 8:**
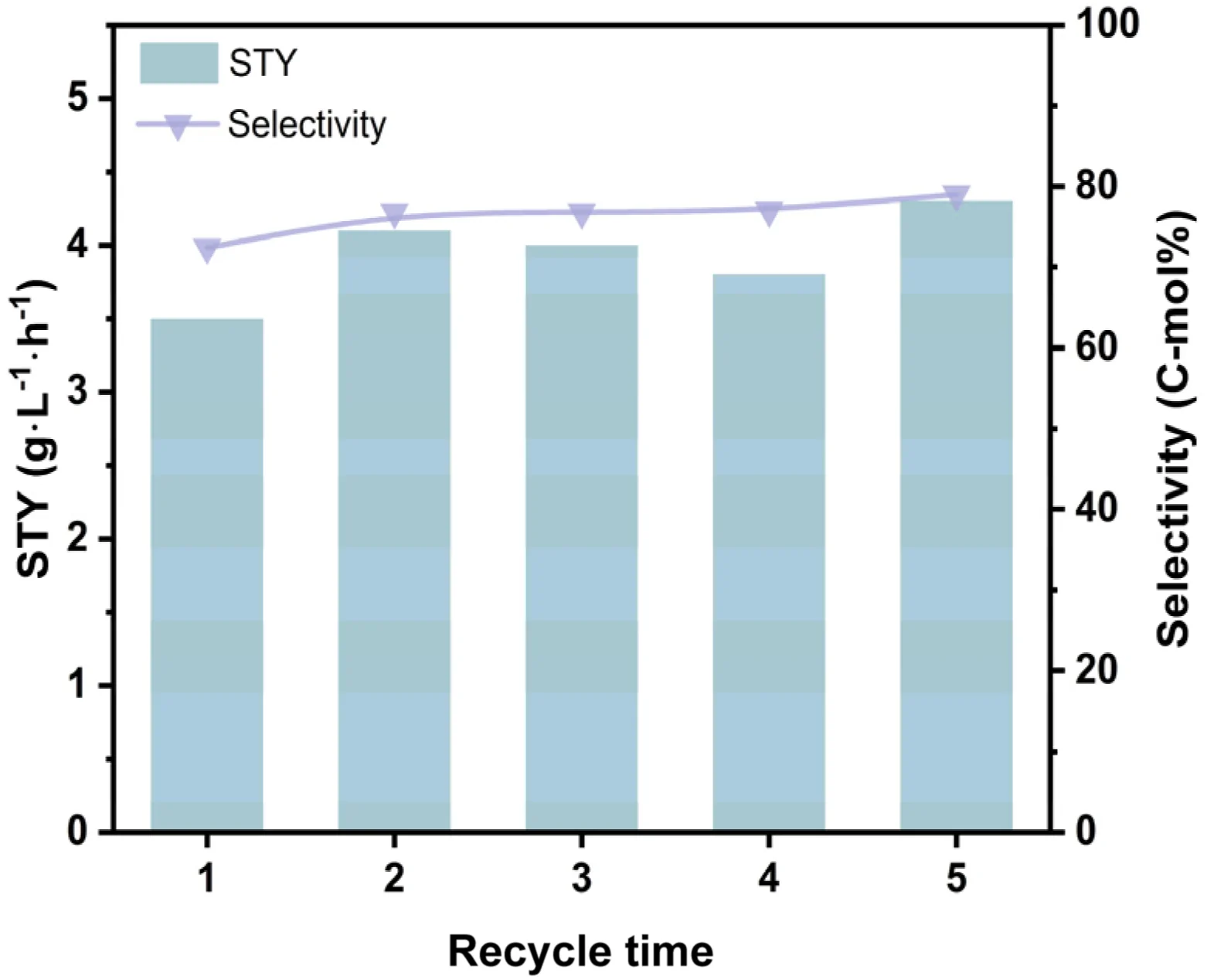
The results of the recycling test. Other reaction conditions were the same as those in entry 1 in Table 1.

## Mechanistic study

### The respective roles of the catalytic components

To clarify the roles of the catalytic components, a series of control experiments were conducted (Table 2). Although RuCl_3_·3H_2_O and CoCl_2_·6H_2_O are cheap and easily available catalyst precursors, without the PPh_3_ ligand, they did not work at all for the synthesis of ethanol *via* reaction of methanol with CO_2_ and H_2_ (entry 1). The PPh_3_ ligand enabled the desired reaction without any pretreatment or pre-activation of RuCl_3_·3H_2_O and CoCl_2_·6H_2_O (entry 2). To understand the role of Ru and/or Co, we started with the control reaction of CO_2_ and H_2_. CO_2_ could be converted into a small amount of CO (0.03 mmol) *via* the RWGS reaction with sole RuCl_3_·3H_2_O, but the catalyst was unstable (entry 3). In the presence of the PPh_3_ ligand, the RuCl_3_·3H_2_O catalyst was stable and more CO (0.09 mmol) was produced (entry 4). The enhanced stability and catalytic activity of the Ru catalyst may be attributed to the synergistic interplay of two effects. First, the σ-donation from PPh_3_ could increase the electron density of the Ru center, which facilitates the RWGS reaction.^48,49^ Second, the dynamic coordination–dissociation behavior of PPh_3_ could generate vacant sites for substrate activation while maintaining catalyst stability.^50^ A considerable amount of the byproduct methane could also be formed by direct hydrogenation of methanol over the Ru catalyst (entry 5). Under similar conditions except for the Co catalyst, a minor amount of ethanol was also detected after the reaction, indicating that the Ru catalyst itself has the ability to promote the formation of ethanol (entry 6). The Co catalyst could not promote the RWGS reaction (Fig. S8). In addition, no ethanol was detected when only Co was utilized under different conditions, confirming that Co could not catalyze ethanol formation independently (entries 7–9). Therefore, in this work, the cooperation of Ru and Co was essential for efficient synthesis of ethanol (entry 10). These findings showed that Ru primarily facilitated hydrogenation, whereas Co was essential for the C–C coupling toward ethanol formation.

**Table 2:** Effect of the different catalytic components^a^

Entry	Catalysts (µmol)		Ligand (µmol)	Substrate (mmol)	Pressure (MPa)			STY	Selectivity (C-mol%)	
	Ru	Co	PPh_3_	Methanol	CO	CO_2_	H_2_		Ethanol	CH_4_
1^b^	10	45	0	10	0	1.3	6.5	0	0	100
2^c^	10	45	40	10	0	1.3	6.5	2.0	55.4	44.6
3^b^^,^^c^	10	0	0	0	0	1.3	6.5	0	0	0
4^c^	10	0	40	0	0	1.3	6.5	0	0	0
5^b^	10	0	0	10	0	0	6.5	0	0	100
6	10	0	40	10	0.2	1.3	6.5	0.2	12.6	86.4
7	0	45	0	10	0.2	0	6.5	0	0	0
8	0	45	40	10	0.2	0	6.5	0	0	0
9	0	45	40	10	0.2	1.3	6.5	0	0	0
10	10	45	40	10	0.2	1.3	6.5	3.5	72.4	27.6

a Reaction conditions: RuCl_3_·3H_2_O and CoCl_2_·6H_2_O were used as catalyst precursors and their dosage was based on the metal, 2.3 mmol LiI, desired amounts of PPh_3_ and methanol, 2 mL NEP, 180 °C, 5 h.

b A black precipitate was observed after the reaction.

c CO was detected after the reaction and the amount of CO followed the order entry 2 (0.64 mmol) > entry 4 (0.09 mmol) > entry 3 (0.03 mmol).

The LiI promoter was also necessary for the target reaction (Table 1, entries 2 and 4). It may activate methanol to form CH_3_I, which then undergoes oxidative addition to active Co species, a preliminary step for C–C bond formation with CO.^42^ CO is another key intermediate for C–C bond formation to produce ethanol. This is the reason why low pressure of CO (0.2 MPa) introduced before the reaction could modulate the reaction performance (Table 2, entries 2 and 10). Notably, in the presence of the PPh_3_ ligand, the synergy of Ru and Co catalyst in this work was not only involved in efficient synthesis of ethanol (entries 6 and 10), but was also involved in the formation of the CO intermediate *via* the RWGS process (entries 2 and 4). An amount of CO as high as 0.64 mmol was detected after the reaction was accelerated by the Ru–Co bimetallic catalyst, although a considerable amount of CO generated *in situ* had been consumed to construct ethanol molecules (entry 2).

As discussed above, the PPh_3_ ligand played multiple and decisive roles in the reaction (Fig. 9). First, the PPh_3_ ligand makes the very cheap and easily available RuCl_3_·3H_2_O and CoCl_2_·6H_2_O precursors directly usable for this challenging reaction; in addition, the dosages of Ru/Co are lower than those reported in most of the previous reports (Table S1). Second, it enhanced the overall catalytic performance of this reaction to a top level, including the activity and selectivity of ethanol production, as well as the catalyst stability. Third, it played important roles in different key catalytic steps of the reaction, such as the RWGS process to generate the CO intermediate, and the C–C bond formation to produce ethanol.

**Fig. 9:**
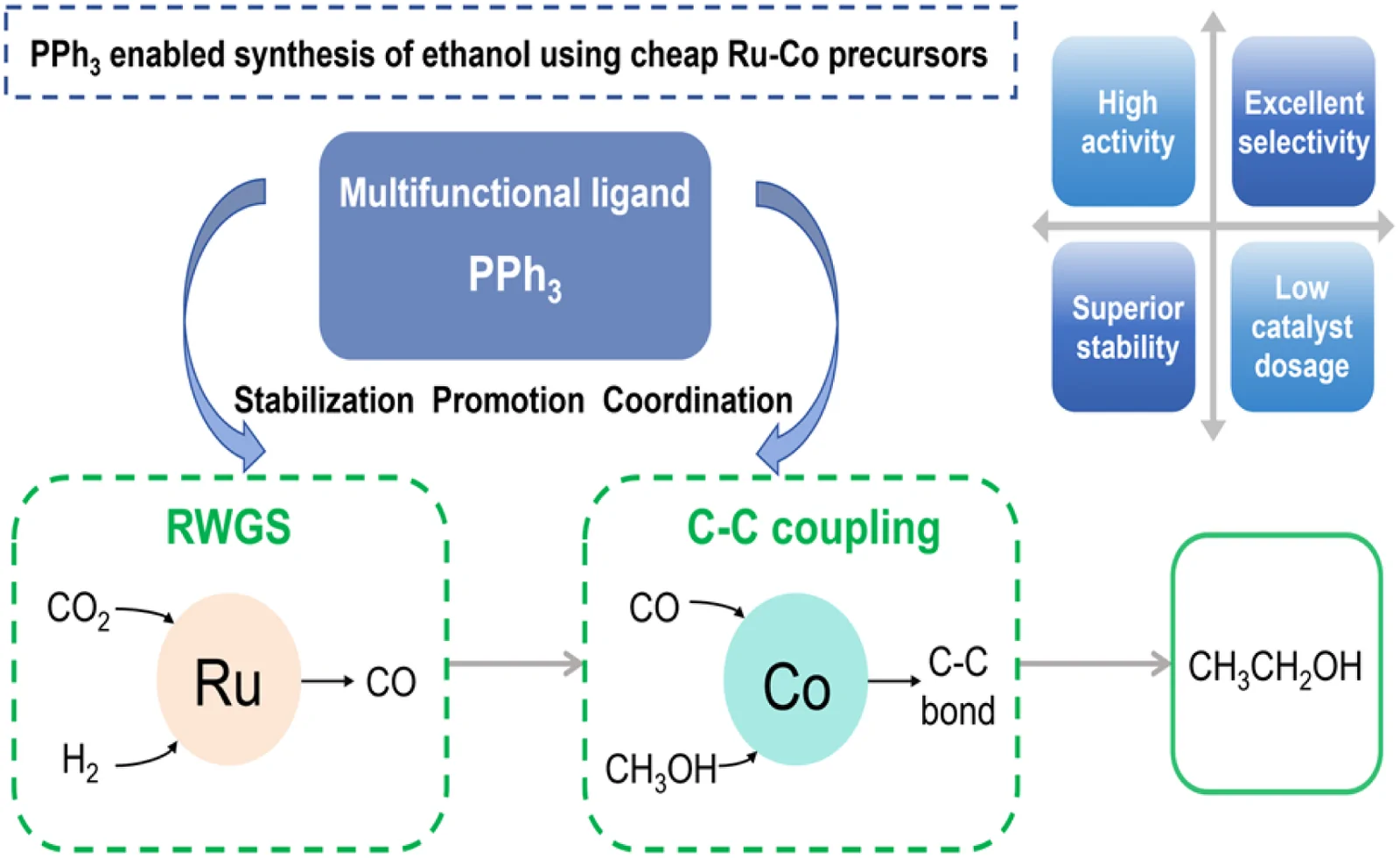
The multifunctional PPh_3_ ligand in the reaction.

### The active species of the Ru and Co catalysts

To learn more about the coordination behavior of PPh_3_ on the Ru–Co catalysts, systematic ^31^P{^1^H} NMR analyses of the reaction solution were carried out and the results are given in Fig. 10. The PPh_3_ ligand in the solution has two distinct signals at around 28 ppm and 22 ppm, indicating the presence of two chemical environments for P. The peak at 28 ppm disappeared when CoCl_2_·6H_2_O was introduced (either alone or together with Ru). In contrast, without a Co precursor, the two peaks were intact whether in the presence of the Ru precursor or not. Further analysis revealed that the Co(i)-PPh_3_ complex displayed a detectable ^31^P signal, whereas no signal was observed for the Co(ii)-PPh_3_ complex, likely due to the paramagnetic nature of Co(ii) (Fig. S9). The paramagnetic Co species may severely broaden or completely suppress certain ^31^P signals *via* fast electron–nuclear relaxation. Therefore, no observable ^31^P peaks appear for paramagnetic cobalt–phosphine complexes. Based on these findings, we proposed that the PPh_3_ ligand primarily coordinated with the Co center. This deduction was further supported by HR-ESI(+)-MS results that the Co center simultaneously coordinated with PPh_3_ and CO during the reaction (Fig. S10).

**Fig. 10:**
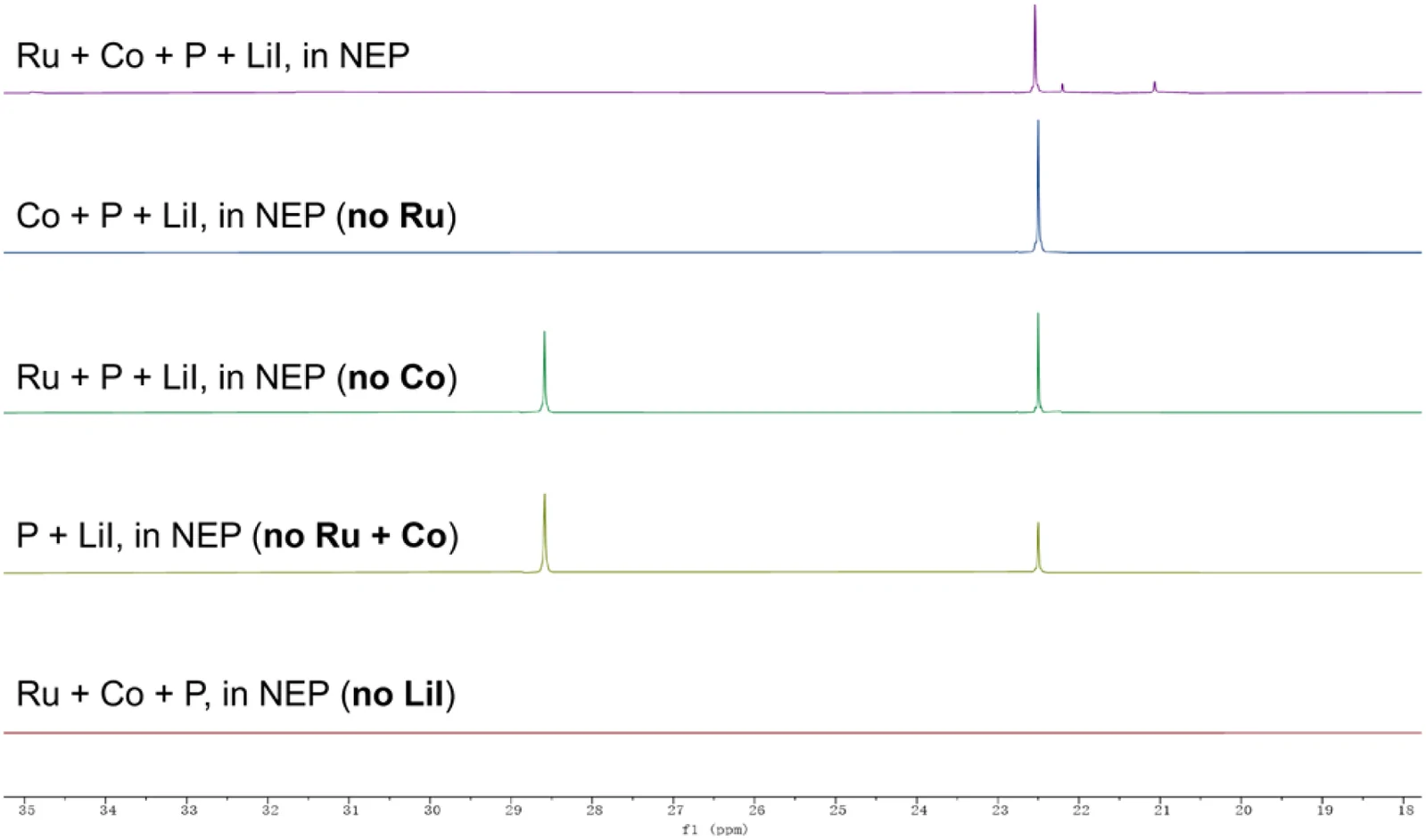
^31^P{^1^H} NMR spectra of the liquid sample after the reaction. Standard reaction conditions were the same as those in entry 1 in Table 1.

The coordination of PPh_3_ with the Co center may greatly enhance the ethanol synthesis (Table S5). Specifically, the combination of 45 µmol Co catalyst and 40 µmol PPh_3_ exhibited even higher performance than the 240 µmol Co catalyst alone under otherwise identical conditions. The electron density of the active Co center (Co*) may be increased through the coordination with PPh_3_, which favours the oxidative addition of CH_3_I to form CH_3_–Co*–I.^51^ Reductive elimination of CH_3_CO in the presence of H_2_ may yield CH_3_CHO, as observed by ESI(+)-MS analysis (Fig. S11 and S12).^52^ This intermediate can then be *in situ* hydrogenated to ethanol by the Ru catalyst (Fig. S13). Additionally, introduction of the PPh_3_ ligand may facilitate the solvent effect by triggering combination of the NEP with the Co cations, which could help to stabilize the Co catalyst (Fig. S11).^53^

During the reaction, the Ru species generated from RuCl_3_·3H_2_O was [Ru(CO)_*x*_I_*y*_]^−^ (*x* = 1,2; *y* = 2,3), as demonstrated by FT-IR and HR-ESI(−)-MS characterization studies (Fig. 11 and S14). It was found that the presence of PPh_3_ did not affect the structure of the Ru species, which agreed with the ^31^P{^1^H} NMR results in Fig. 10.

**Fig. 11:**
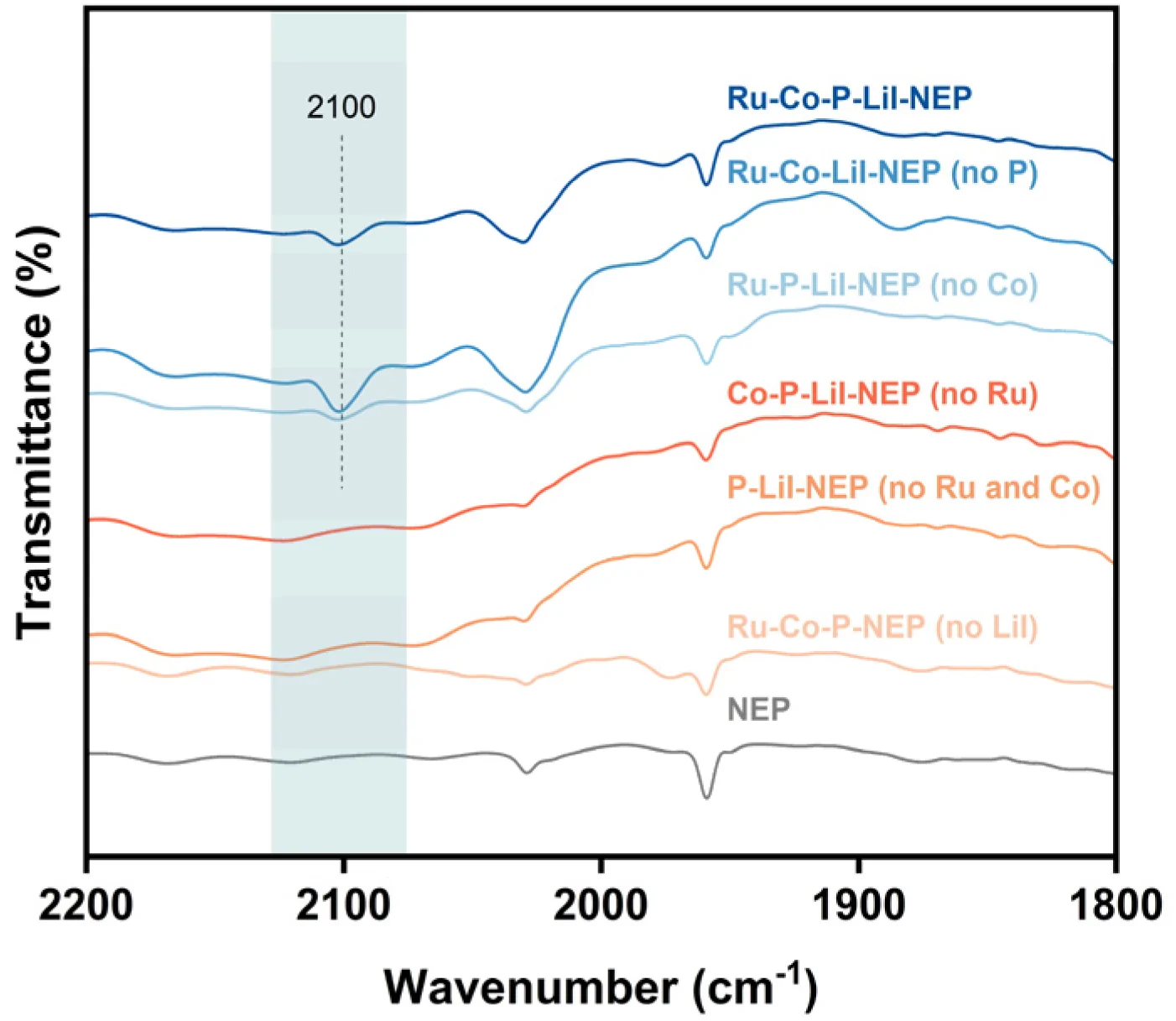
FT-IR spectrum of the liquid sample after the reaction. Reaction conditions were the same as those in entry 1 in Table 1.

In the FT-IR spectra, the peak observed at around 2100 cm^−1^ is ascribed to the carbonyl stretching vibration of CO coordinated to the Ru center (Fig. 11). However, no evident peak for Co–CO was seen in the FT-IR spectra. We further analysed the solution of Co_2_(CO)_8_ in NEP and toluene, respectively, but no peak was observed in either (Fig. S15). It has been reported that the cobalt carbonyl complexes in solution undergo fast isomerization and solvent–solute dynamic friction, and the rapid interconversion between isomers shortens vibrational lifetimes, causing severe IR peak broadening and disappearance.^54^ It is worth noting that LiI is necessary for the formation of the above Ru and Co species during the reaction.

To identify the active catalytic species, we directly utilized the proposed Ru and Co intermediates (Table S6). We successfully synthesized the Ru intermediates ([Ru(CO)I_3_]^−^, [Ru(CO)_2_I_3_]^−^, and [RuI_3_]^−^) as identified by HR-ESI(−)-MS (Fig. S16). When the synthesized Ru intermediates were used as the catalyst, enhanced catalytic performance was observed (entry 2), confirming the involvement of these Ru species in the catalytic cycle. Furthermore, replacement of CoCl_2_·6H_2_O with Co(PPh_3_)_2_Cl_2_ or Co(PPh_3_)_3_Cl revealed that the PPh_3_ ligand helped to gain higher ethanol selectivity. In addition, Co(PPh_3_)_2_Cl_2_ afforded superior catalytic activity, whereas Co(PPh_3_)_3_Cl gave lower activity (entries 3 and 4). This trend was consistent with the proposed paramagnetic nature of the active Co intermediate.

### The carbon source of ethanol

To gain insight into the exact transformation pathway of CH_3_OH and CO_2_ during the catalytic reaction, we performed isotope tracer experiments using ^13^CH_3_OH and ^13^CO_2_ as labelled reactants, respectively, and analysed the resulting liquid and gas products *via* GC-MS (Fig. S17–S20). In the ^13^CH_3_OH-labeled system, the product ethanol was identified as ^13^CH_3_CH_2_OH, confirming that the methyl group of methanol was directly incorporated into the methyl moiety of ethanol. Meanwhile, ^13^CH_4_ was detected in the gas phase, indicating that a portion of the methanol was converted into methane. Notably, the CO byproduct remained unlabelled, suggesting that CO was not generated from methanol under these conditions.

In the ^13^CO_2_-labelled system, ethanol appeared as CH_3_^13^CH_2_OH, demonstrating that the carbon from CO_2_ selectively occupied the methylene (–CH_2_–) position of ethanol. Additionally, ^13^CO was observed in the gas phase, verifying that the ^13^CO_2_ was first reduced to CO. In contrast, the methane product was unlabelled CH_4_, confirming that methane formation relied exclusively on methanol as the carbon source rather than CO_2_. These results clearly delineate the distinct carbon flow in the PPh_3_–Ru–Co–LiI catalytic system: methanol serves as the carbon donor for the ethanol methyl group and methane, while CO_2_ contributes specifically to the ethanol methylene group and CO intermediate (Fig. 12). This isotopic evidence provides direct mechanistic insights into the selective coupling of methanol and CO_2_ for ethanol synthesis.

**Fig. 12:**
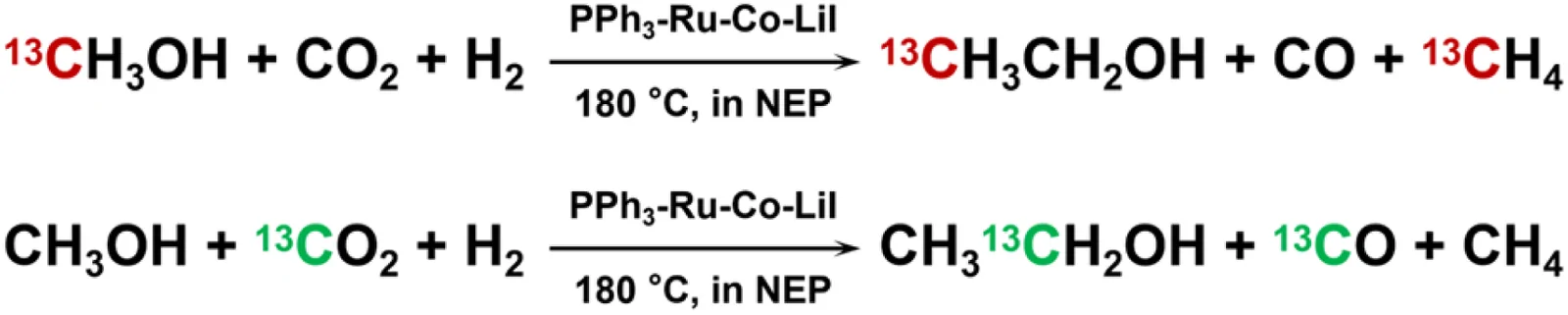
The transformation path of CH_3_OH and CO_2_ according to the ^13^C labelling experiments.

### Mechanistic discussion

Based on the above discussion, we proposed a possible mechanism for ethanol synthesis *via* reaction of methanol with CO_2_ and H_2_ (Fig. 13). First, methanol was converted to CH_3_I in the presence of LiI (step 1, Fig. S21),^42^ which then added oxidatively to the active Co species (complex a, denoted as [Co–P]*) to form CH_3_–[Co–P]*–I (complex b) (step 2). Second, CO was generated *in situ via* Ru-catalyzed CO_2_ hydrogenation (step 3). Third, CO inserted into the [Co–P]*–CH_3_ bond of complex b, forming a new C–C bond and affording CH_3_CO–[Co–P]*–I (complex c) (step 4). This was followed by the oxidative addition of H_2_ to complex c, yielding complex d (step 5). Reductive elimination of acetaldehyde from complex d regenerated the active Co species (complex a) and released HI (step 6). The *in situ* generated acetaldehyde was quickly hydrogenated to ethanol by the active Ru species (step 7). Finally, the HI produced in step 6 was consumed by reaction with LiOH to form LiI (step 8). Then the next catalytic cycle began.

**Fig. 13:**
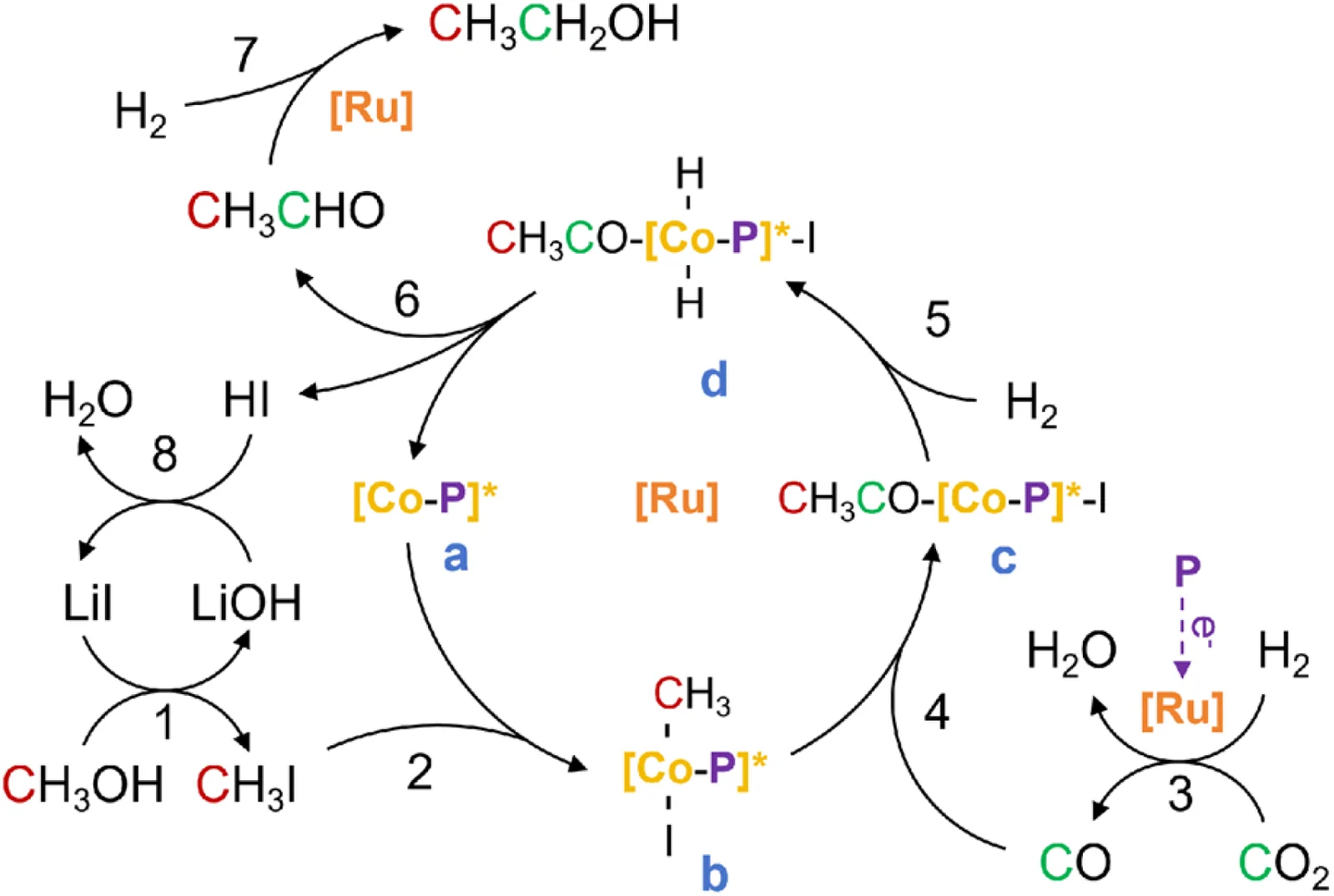
The proposed reaction mechanism.

## Conclusion

In summary, we developed a highly efficient and economical homogeneous catalytic system for the direct synthesis of ethanol from CO_2_, methanol, and H_2_, using cheaper and readily available RuCl_3_·3H_2_O and CoCl_2_·6H_2_O precursors enabled by the PPh_3_ ligand. Under optimized conditions, the system achieved an ethanol STY of 4.4 g L^−1^ h^−1^, TOF of 23.2 h^−1^, and ethanol selectivity of 85.6 C-mol%, representing a notable advance compared with reported homogeneous catalysts for ethanol synthesis *via* CO_2_ hydrogenation. *N*-ethyl-2-pyrrolidone (NEP) was identified as the suitable solvent, owing to its unique cyclic amide structure, good solubility of LiI, and favourable solvation for gaseous reactants. Reaction parameter studies revealed that 180 °C, a CO/CO_2_/H_2_ pressure of 0.2/1.3/8.5 MPa, and a methanol/NEP volume ratio of 0.4/2.0 gave the best performance. A series of control experiments verified the major roles of each component: Ru drives CO_2_ hydrogenation and the final acetaldehyde hydrogenation step; Co enables C–C bond formation in the presence of Ru; LiI promotes methanol activation and methyl-cobalt species formation; the PPh_3_ ligand stabilizes the Ru–Co catalyst and improves activity and selectivity, while suppressing CH_4_ formation. In the presence of PPh_3_, close synergy between Ru and Co catalysts was observed in accelerating the RWGS and C–C coupling steps. The system also exhibited excellent recyclability with consistent performance over five runs, demonstrating outstanding stability. ^13^C isotope tracer experiments clearly established the carbon-flow pathway: the methyl group of ethanol originates from methanol, while the methylene group comes from CO_2_, which is first converted to CO *via* the RWGS reaction. Mechanistic characterization revealed that PPh_3_ preferentially coordinates to cobalt centers, modulating electron density and facilitating oxidative addition and CO insertion steps. The active Ru species were identified as [Ru(CO)_*x*_I_*y*_]^−^ complexes. The proposed reaction mechanism comprises several sequential steps: LiI promoted methanol activation to generate CH_3_I, Ru-catalyzed CO_2_ hydrogenation to CO, C–C coupling between CO* and 
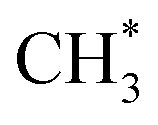
 over the Co active center in the presence of the Ru catalyst, and final Ru-catalyzed hydrogenation of acetaldehyde to ethanol. This study establishes a practical, low-cost, and highly selective route for CO_2_ upgrading to ethanol by coupling industrial methanol with CO_2_ and H_2_. We would like to mention that the results in this work are still preliminary for industrial development, and further kinetic studies are to be carried out.

## Author contributions

Jia Guo: investigation, methodology, formal analysis, writing – original draft, visualization. Ying Wang, Xianyu Meng, Ziyu Li, and Shenggui He: data curation, methodology, formal analysis. Jie Li and Hongxing Wang: resources, validation, discussion. Chenglong Yu and Zhuo Zhang: formal analysis, discussion. Qingli Qian and Buxing Han: conceptualization, methodology, supervision, writing – review & editing, funding acquisition.

## Conflicts of interest

There are no conflicts to declare.

## Supplementary Material

SC-017-D6SC02731H-s001

## Data Availability

The data supporting this article have been included as part of the supplementary information (SI). Supplementary information: experimental methods, figures, and tables. See DOI: https://doi.org/10.1039/d6sc02731h.
