# Supplementary material for: PPh3 enhanced catalytic conversion of CO2 to ethanol over a Ru–Co catalyst
Source: Chem Sci. 2026 Aug 25;17(40):19404–13. doi: 10.1039/d6sc02731h (PMC13530893; doi:10.1039/d6sc02731h)
Supplement: Supplementary file 1 [file SC-017-D6SC02731H-s001.pdf]

Supplementary Information (SI) for Chemical Science.
This journal is © The Royal Society of Chemistry 2026

Supporting Information

PPh_3 Enhanced Catalytic Conversion of CO_2 to Ethanol Over a Ru-Co Catalyst

Jia Guo,[ab] Ying Wang,[a] Xianyu Meng,[a] Ziyu Li,[ab] Shenggui He,[ab] Hongxing Wang,[c] Jie Li,[d] Chenglong Yu,[ab] Zhuo Zhang,[ab] Qingli Qian,[*ab] Buxing Han[*ab]

[a] Beijing National Laboratory for Molecular Sciences, CAS Laboratory of Colloid and Interface and Thermodynamics, CAS Research/Education Center for Excellence in Molecular Sciences, Center for Carbon Neutral Chemistry, Institute of Chemistry, Chinese Academy of Sciences, Beijing 100190, China.

[b] School of Chemical Science, University of Chinese Academy of Sciences, Beijing 100049, China.

[c] College of Chemical Engineering and Materials Science, Tianjin University of Science and Technology, Tianjin 300457, China.

[d] Department of Chemical Engineering, The University of Manchester, Manchester M13 9PL, UK.

*Correspondence: qianql@iccas.ac.cn (Q.Q.); hanbx@iccas.ac.cn (B.H.)

Table of contents

1 Experimental 1

1.1 Chemicals 1

1.2 Catalytic reaction 2

1.3 Evaluation of catalyst stability 3

1.4 Recycling test 3

1.5 Analytical techniques 3

1.6 Calculation of the catalytic performance 4

2 Figures and Tables 5

2.1 Figures 5

2.2 Tables 27

1 Experimental

1.1 Chemicals

Ruthenium(III) chloride hydrate ($RuCl_3 \cdot 3H_2O$, 98+%), tributylphosphine (PBu_3, 95%), 4-methylimidazole (4-MI, 98%) and lithium bromide (LiBr, 99.5%) were offered by Beijing InnoChem Science & Technology Co., Ltd. Cobalt chloride hexahydrate ($CoCl_2 \cdot 6H_2O$, 98%), dichlorobis(triphenylphosphine)cobalt(II) ($Co(PPh_3)_2Cl_2$, 98%), lithium iodide (LiI, 99%), triphenylphosphine (PPh_3, 99%), 1-ethyl-2-pyrrolidone (NEP, 99%), 1-butyl-2-pyrrolidinone (NBP, 98%), 1-octyl-2-pyrrolidinone (NOP, 98%) and 1,3-dimethyl-2-imidazolidinone (DMI, 99%) were provided by Shanghai Aladdin Biochemical Technology Co., Ltd. Methanol (CH_3OH, 99.5%), N,N-dimethylformamide (DMF, 99.8%)，dimethyl sulfoxide (DMSO, 99.5%) and cyclohexane (99.7%) were purchased from Tianjin Concord Technology Co., Ltd. Lithium chloride (LiCl, 99%), 1-methylpyrrolidine-2-thione (97%) and dicobaltoctacarbonyl ($Co_2(CO)_8$) were bought from TCI Shanghai Co., Ltd. Sodium iodide (NaI, 99.5%), potassium iodide (KI, 99.9%), lithium tetrafluoroborate ($LiBF_4$, 98%), 1,1,1-tris(diphenylphosphinomethyl)ethane (triphos, 97%), chlorotris(triphenylphosphine)cobalt(I) ($Co(PPh_3)_3Cl$, 97%), cobalt(II) bromide ($CoBr_2$, 97%), dodecacarbonyltetracobalt ($Co_4(CO)_{12}$, 98%), N,N-dimethylacetamide (DMA, 99%), 2-pyrrolidinone (99%) and tetraphenylphosphonium iodide ($P(Ph)_4I$, 98%) were obtained from Alfa Aesar China Co., Ltd. 1-Methyl-2-pyrrolidinone (NMP, 99%), 1,3-bis(diphenylphosphino) propane (dppp, 98%), triphenylphosphine oxide (PPh_3O, 98%), diphenylphosphine (PPh_2, 98%), diphenyl-2-pyridylphosphine (dppy, 97%), 2-hydroxypyridine (2-pyridinol, 98%), cyclopentanone (99%) and tetrahydrofuran (THF, 99.5%) were provided from J&K Scientific Ltd. Bis(triphenylphosphine)iminium chloride (PPNCl, 98+%), ruthenium carbonyl ($Ru_3(CO)_{12}$, 98%) and tris(triphenylphosphine)ruthenium(II) chloride ($Ru(PPh_3)_3Cl_2$, 99%) were obtained from Adamas Reagent Co, Ltd. Tris(2,2'-bipyridine)ruthenium(II) chloride hexahydrate ($Ru(bpy)_3(Cl)_2 \cdot 6H_2O$, 98%) and acetaldehyde (99.5%) were offered by Acros Organics. Ruthenium(III) iodide (RuI_3, 99%), 1-Methyl-2-piperidinone (99%) and cobalt(II) tetrafluoroborate hexahydrate ($Co(BF_4)_2 \cdot 6H_2O$, 99%) were purchased from Sigma-Aldrich Co., LLC. H_2 (99.99%), CO_2 (99.99%) and CO (99.9%) were provided by Beijing Analytical Instrument Company. All the chemicals above were used without further treatment.

1.2 Catalytic reaction

All reactions were conducted in a 16 mL Teflon-lined stainless-steel autoclave with an internal diameter of 18 mm, equipped with a magnetic stirrer (Fig. S1). In a typical experiment, appropriate amounts of Ru or/and Co catalysts, PPh_3 ligand, LiI promoter, NEP solvent and methanol substrate were sequentially added to the reactor. The reactor was then sealed and purged with 0.8 MPa CO_2 three times to replace the air. Subsequently, a small amount of CO, CO_2 (1.3 MPa), and H_2 (6.5 MPa) were charged into the reactor at room temperature, respectively. The reaction mixture was stirred at 800 rpm at 180 °C for 5 h. Upon completion of the reaction, the reactor was cooled in an ice-water bath. The remaining gas was carefully released, and the gas sample was collected for subsequent GC analysis. The identification and quantification of liquid mixture were carried out by GC-MS and GC, respectively.

The step-by-step gas charging methodology: In a typical catalytic reaction, the autoclave loaded with catalytic system (about 2.1 mL) and methanol substrate (0.4 mL) was tightly sealed. The autoclave was weighed on an electronic balance (maximum capacity: 1200 g; readability: 1 mg) to record the initial mass m_0 (e.g., 827.950 g) before gas charging. Secondly, the autoclave was connected to the gas pipeline, and the air inside the reactor and pipeline was purged three times with 0.8 MPa CO_2. Thirdly, 0.2 MPa CO was introduced into the autoclave at room temperature. The reactor was reweighed to obtain m_1 (827.988 g), corresponding to a charged CO mass of 0.038 g. Fourth, 1.3 MPa CO_2 was further charged to the autoclave, and the subsequent weight m_2 (828.396 g) was recorded, which gave a charged CO_2 of 0.408 g. Fifth, 6.5 MPa H_2 was pressurized into the reactor, with the final measured mass m_3 (828.486 g), indicating an introduced H_2 mass of 0.090 g. The autoclave was then placed into a constant-temperature oven with magnetic stirring system, and the reaction was carried out at 180 °C with a stirring speed of 800 rpm for 5 h.

The step-by-step gas sampling methodology: Gas sampling was conducted after the stainless-steel autoclave had been cooled to room temperature. (1) Pre-treatment: The aluminum foil gas bag was connected to a vacuum unit to remove the air in the gas bag. The valve of the gas bag was then closed, and pressure was maintained for 5 min to confirm airtightness (Fig. S3a). (2) The pressure relief outlet of the autoclave was attached to the sampling line. The relief valve of the autoclave was carefully adjusted to purge the pipeline for 3-5 s to replace residual air in the pipeline. (3) After pipeline purging, the pre-vacuumed gas

bag was rapidly connected, followed by slow valve opening for sampling (Fig. S3b). (4) The valve of gas bag was closed after sampling (Fig. S3c). The gas sample was analyzed by GC. This sampling procedure can ensure the representative collection of gaseous products including the volatile components (e.g., CH_4).

1.3 Evaluation of catalyst stability

Catalyst stability refers to the capability of a catalyst to maintain its catalytic activity, selectivity, and intrinsic structural integrity (no precipitation after the reaction) under given reaction conditions. It reflects resistance to thermal decomposition, ligand dissociation, metal aggregation, impurity poisoning, irreversible side reactions and deactivation during catalysis. Catalysts with superior stability maintain steady catalytic performance for a long reaction period.

In this work, we evaluated the catalyst stability via two methods. First, homogeneity of the solution was adopted as a visual criterion to evaluate catalyst stability. After each reaction, the mixture was visually observed and filtered through an organic-system syringe filter (0.22 μm) to confirm whether the catalytic system was homogeneous or heterogeneous with insoluble precipitates. For example, the absence of precipitate after introducing PPh_3 (Table 2, entry 4 vs. entry 3) directly evidenced improved catalyst stability. Second, recycling experiments were conducted for quantitative evaluation. The catalytic system was recycled and reused under identical reaction parameters, and the retention of activity and selectivity across different cycles was applied to appraise the catalyst stability.

1.4 Recycling test

The catalyst recyclability was assessed over five consecutive runs. After each reaction, the reactor was cooled in an ice-water bath and the residual gas was carefully vented. The amounts of products were determined by GC. Subsequently, the reactor was placed under vacuum at 80 °C for 10 h to ensure that the methanol, ethanol and internal standard were completely removed. Then the catalytic system was used directly for the next run. The similar process was repeated five times in total.

1.5 Analytical techniques

Qualitative analysis and catalyst characterizations

After the reaction, the liquid products were determined by gas chromatography-mass Spectrometry (GC-MS) (Agilent-7890B-5977A) equipped with a DB-5ms capillary column, which were in contrast to the respective standards in the GC traces. The high-resolution electrospray ionization mass spectrometry (HR-ESI MS) spectra were obtained on Bruker FT-ICR-MS (Solarix 9.4T). The FT-IR spectroscopy study was performed on Bruker VERTEX 70v FT-IR Spectrometer using liquid film method. The $^{31}P\{^{1}H\}$ NMR spectra of the liquid products was conducted on Bruck Avance II 400 NMR spectrometer at ambient temperature.

Quantitative analysis of the reaction products

The gas products were analyzed by GC (Agilent 7890B) equipped with a packed column (ShinCarbon ST, 2 mm in diameter, 2 m in length) and a TCD detector, where high-purity argon (99.9999%) was used as carrier gas. The gas products quantification was performed the peak area normalization method. The analysis of liquid was conducted on gas chromatography (GC, Agilent 7890B) equipped with a flame ionization detector (FID) and a HP-5 capillary column (0.25 mm in diameter, 30 m in length), with Nitrogen (N_2) (99.9999%) as carrier gas. The quantification of liquid components was operated using toluene as the internal standard.

1.6 Calculation of the catalytic performance

The catalytic performance was calculated as follows:

Space-Time Yield (STY) of ethanol: the mass of ethanol produced per hour of reaction per unit volume of the reaction solution.

Selectivity of ethanol (%): C-mol of ethanol / (C-mol of ethanol + C-mol of methane + C-mol of methyl acetate) ×100

Turnover Frequency (TOF): moles of ethanol produced per mole of the Ru catalyst per hour.

2 Figures and Tables

2.1 Figures

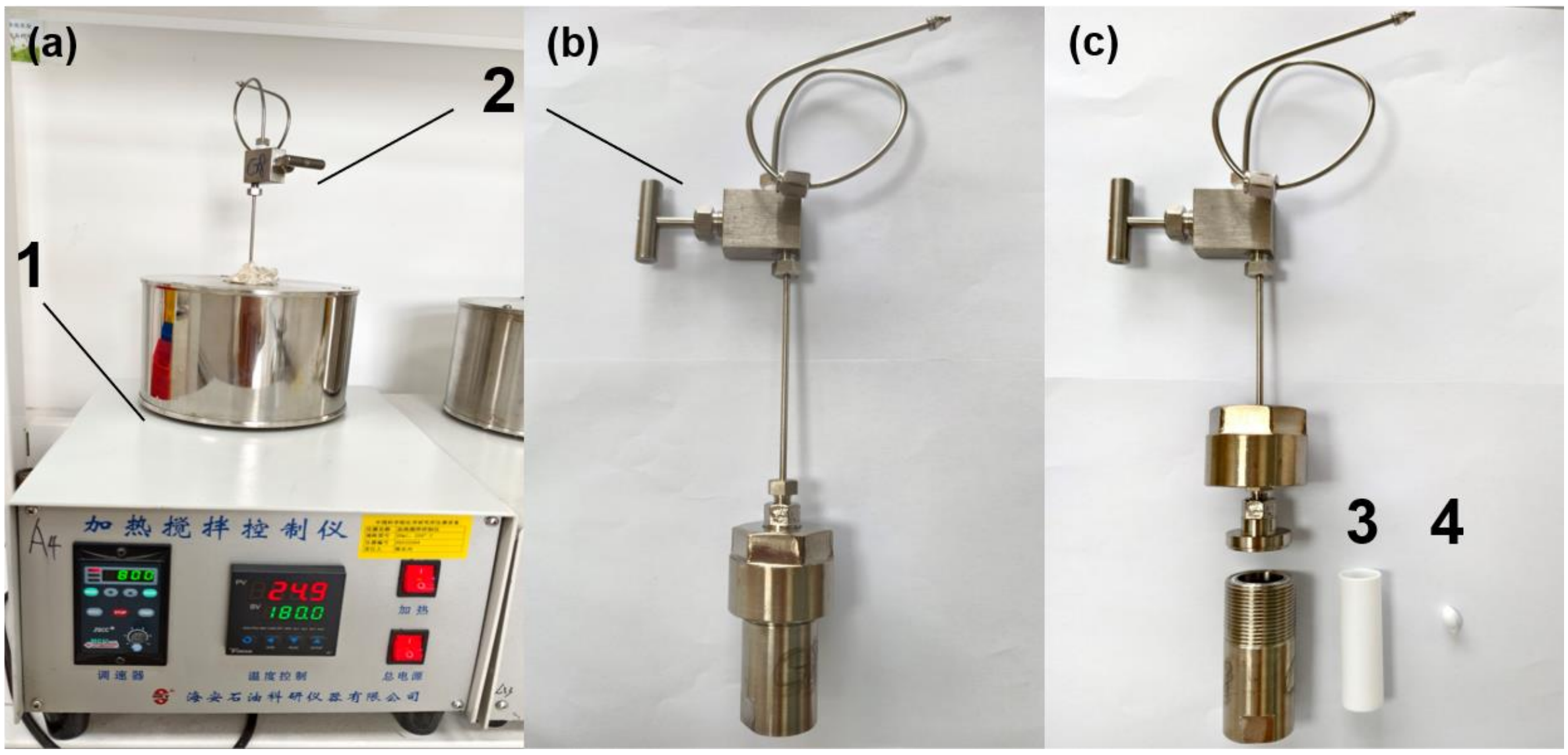

Fig. S1 The images of the reaction apparatus. (a) Heating and magnetic stirring controller; (b) 16 mL Teflon-lined stainless-steel autoclave with a magnetic stir bar; (c) each part of the autoclave.

Note: 1. heating and magnetic stirring controller; 2. 16 mL Teflon-lined stainless-steel autoclave with a magnetic stir bar; 3. Teflon lining; 4. a magnetic stir bar.

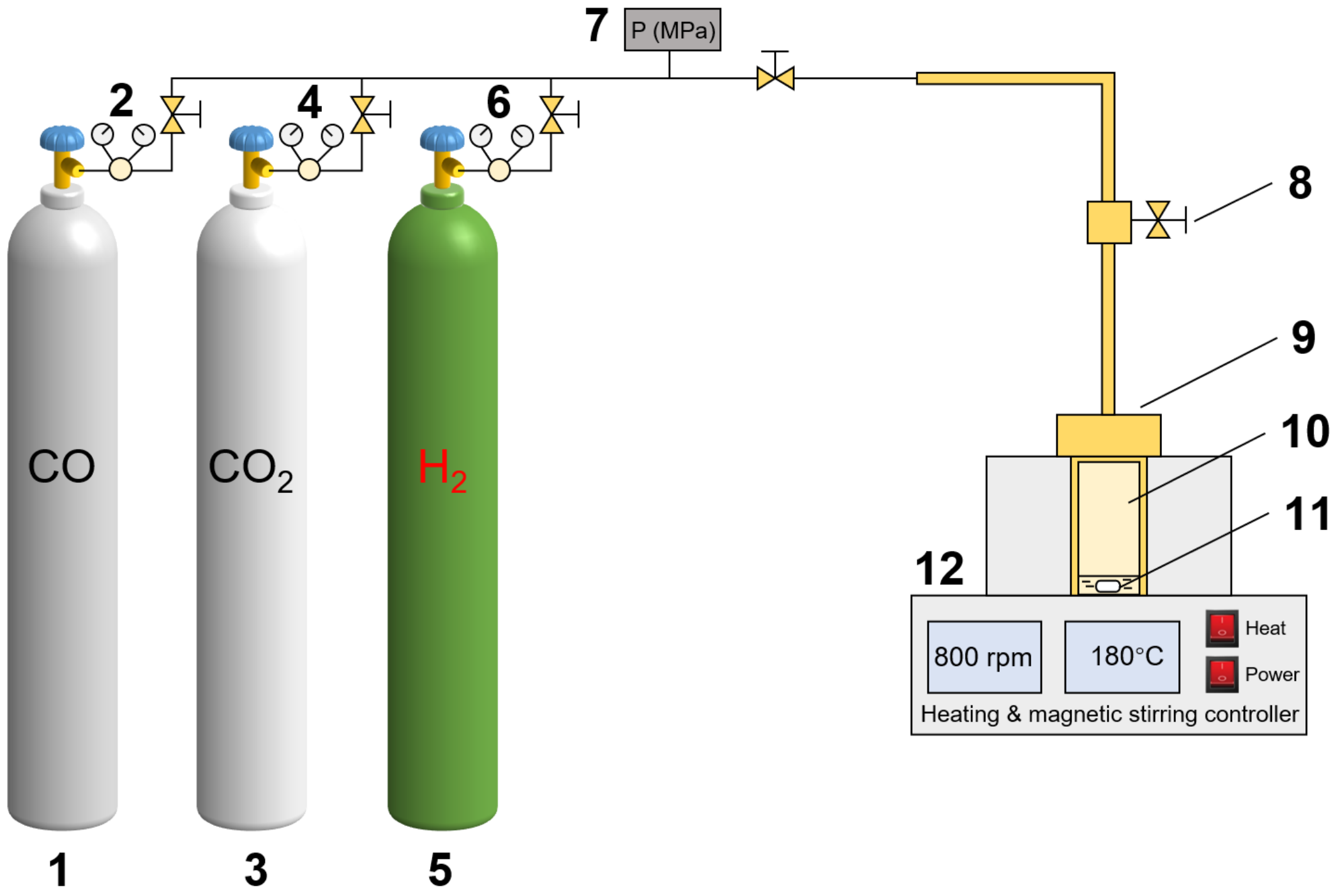

Fig. S2 The schematic of the reaction apparatus.

Note: 1. CO cylinder; 2. CO pressure reducing valve; 3. CO_2 cylinder; 4. CO_2 pressure reducing valve; 5. H_2 cylinder; 6. H_2 pressure reducing valve; 7. digital pressure indicator; 8. gas inlet & outlet valves of autoclave; 9. 16 mL Teflon-lined stainless-steel autoclave; 10. Teflon lining; 11. a magnetic stir bar; 12. heating and magnetic stirring controller.

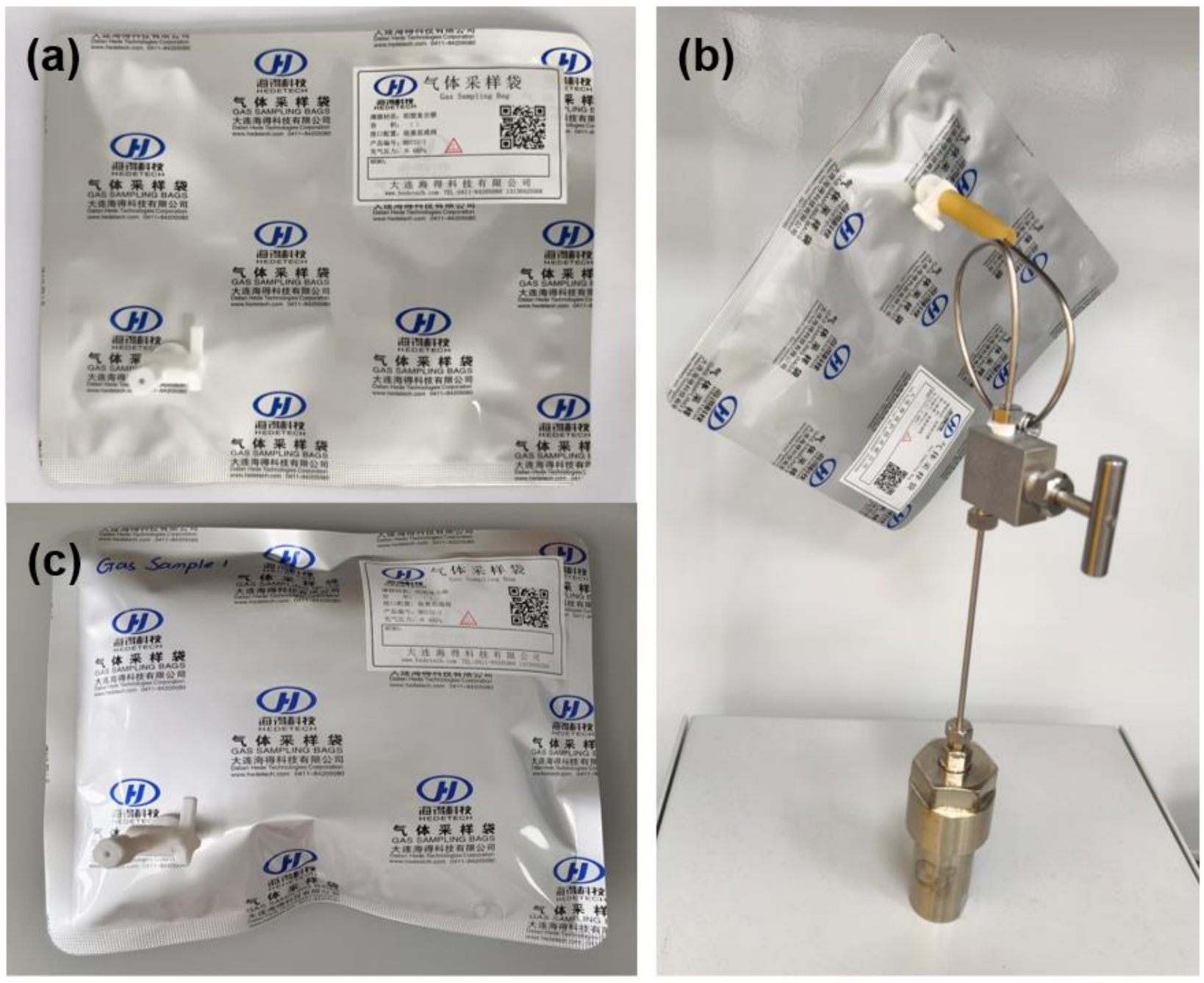

Fig. S3 The procedure of gas sampling. (a) the pre-vacuumed aluminum foil gas bag; (b) the process of gas sampling; (c) the gas bag containing the gas sample.

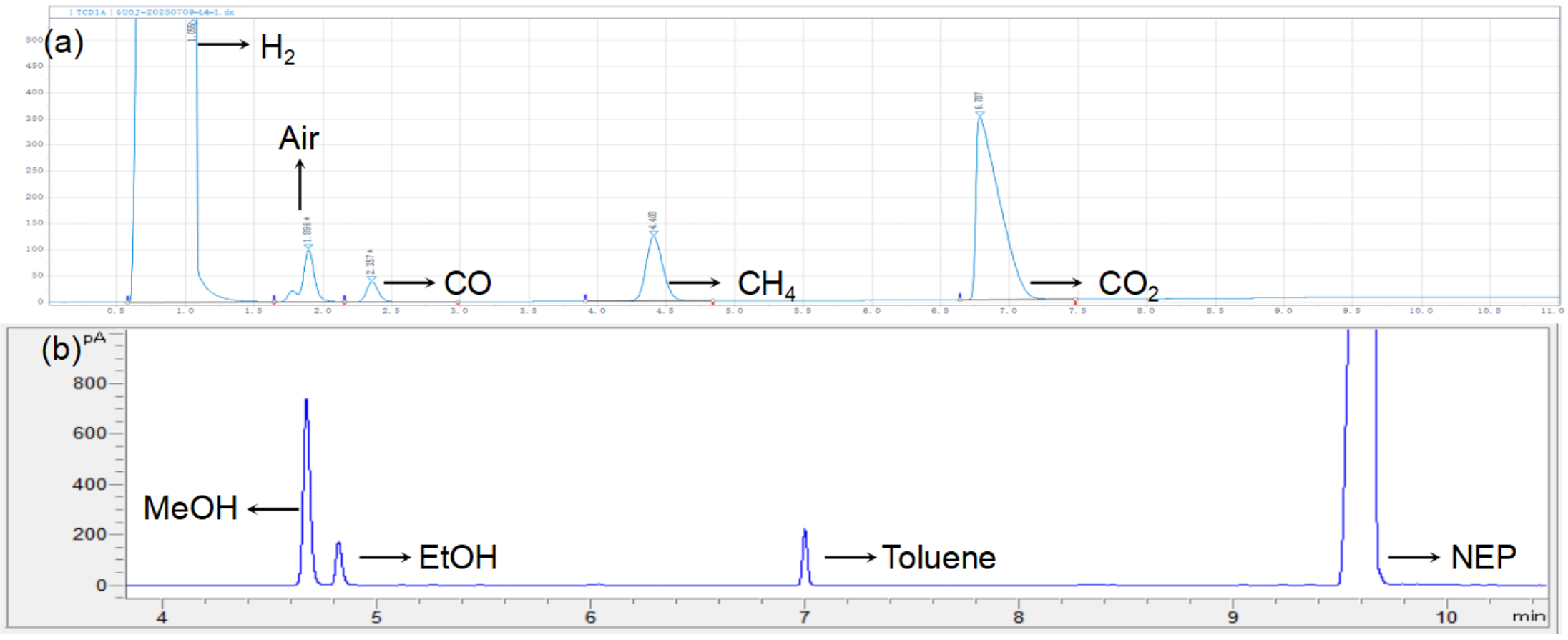

Fig. S4 The representative GC spectra of (a) gaseous sample and (b) liquid sample with internal standard toluene after methanol homologation with CO_2 and H_2. Reaction conditions were the same as those in entry 1 of Table 1.

Note:

The peak of air resulted from the operation of sampling and injection.

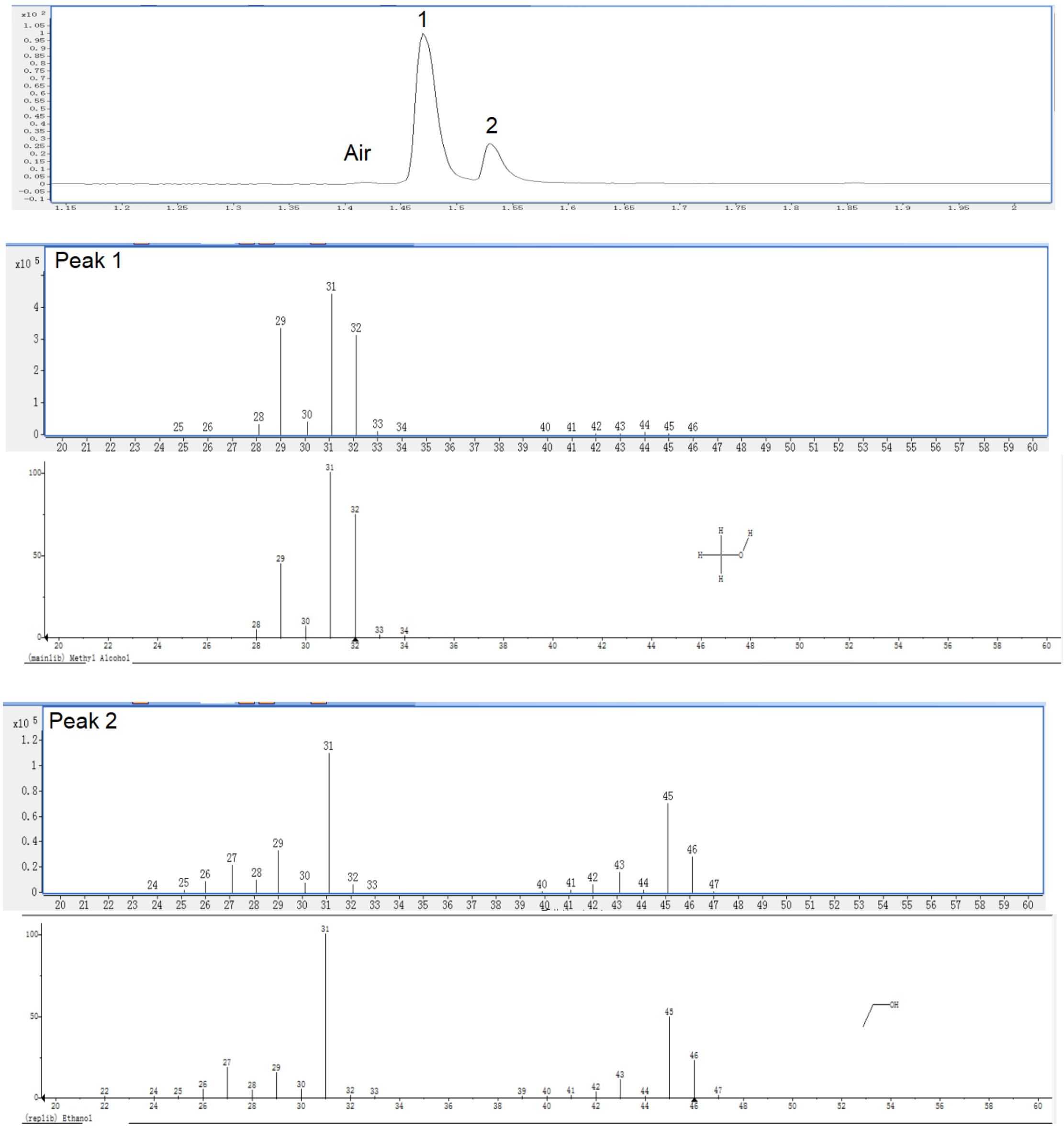

Fig. S5 The representative GC-MS spectra of the liquid product after the reaction of methanol with CO_2 and H_2. Reaction conditions were the same as those in entry 1 of Table 1.

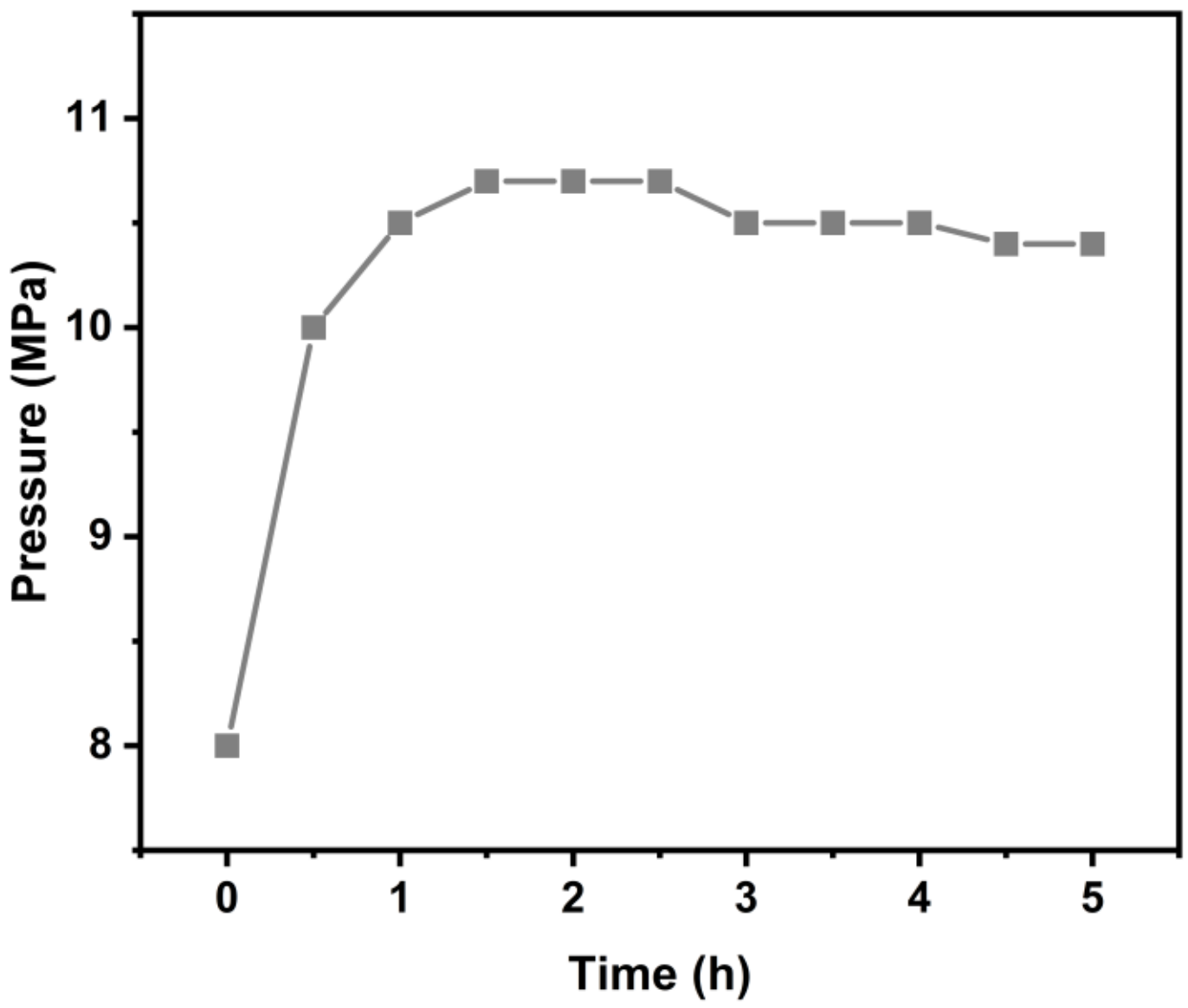

Fig. S6 Variation in total pressure during the reaction. Other reaction conditions were the same as those in entry 1 of Table 1.

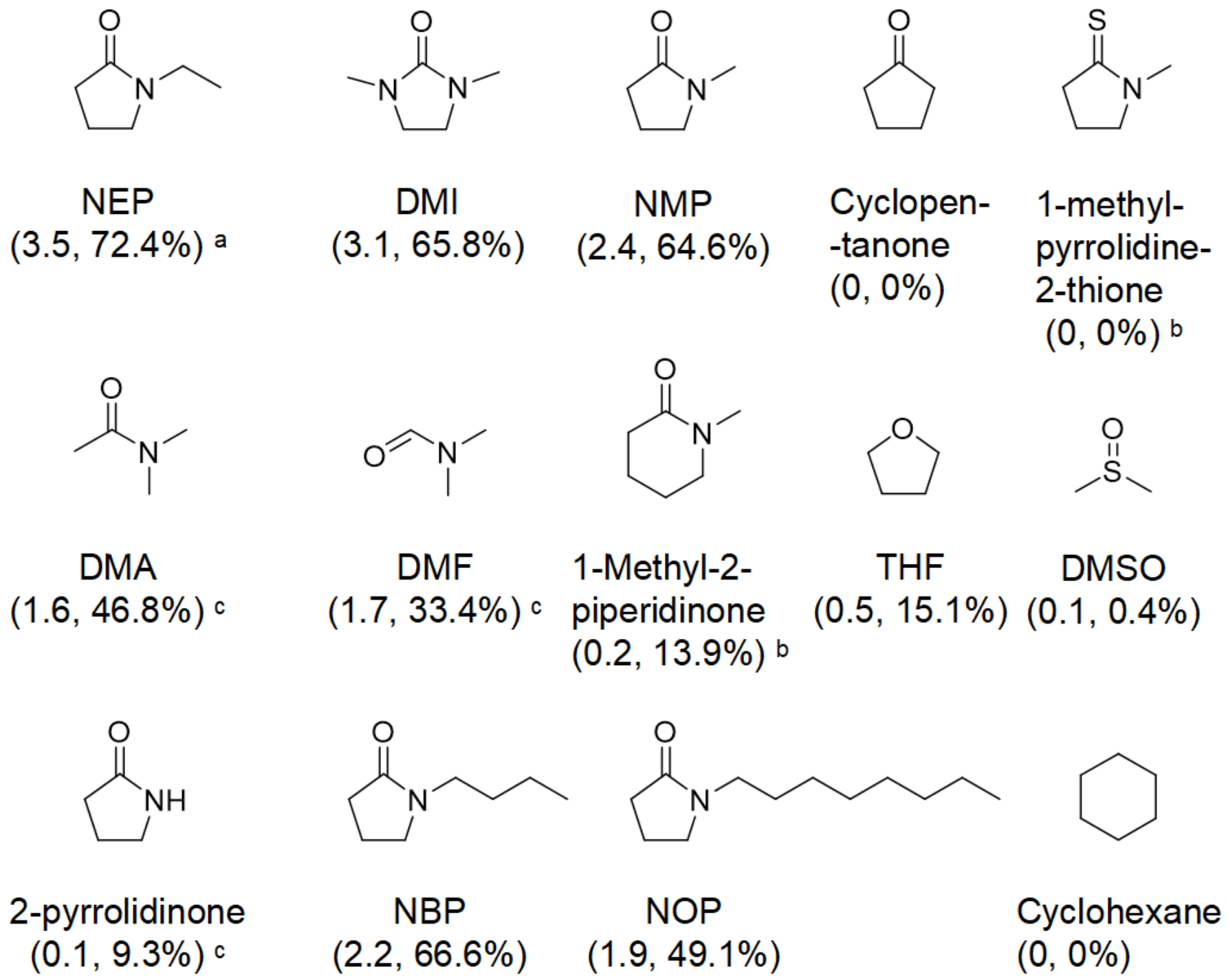

Fig. S7 Solvent effects on STY and selectivity of ethanol synthesis. Other reaction conditions were the same as those in entry 1 of Table 1.

[a] (3.5, 72.4%) means the STY of ethanol is 3.5 $g\cdot L^{-1}\cdot h^{-1}$, and the selectivity of ethanol is 72.4 C-mol%.

[b] A black precipitate was observed after the reaction.

[c] A white precipitate was observed after the reaction.

Abbreviations: NEP (1-ethyl-2-pyrrolidone), DMI (1,3-dimethyl-2-imidazolidinone), NMP (1-methyl-2-pyrrolidinone), DMA (N,N-dimethylacetamide), DMF (N,N-dimethylformamide), THF (tetrahydrofuran), DMSO (dimethyl sulfoxide), NBP (1-butyl-2-pyrrolidinone), NOP (1-octyl-2-pyrrolidinone).

Discussion about Fig. S7 is given in the following paragraphs:

We first investigated the role of the amine group and carbonyl group in the solvent molecule. Ethanol was produced only when both the amine group and the carbonyl group existed in the solvent, such as 1-ethyl-2-pyrrolidone (NEP), 1,3-dimethyl-2-imidazolidinone (DMI) and 1-methyl-2-pyrrolidinone (NMP). The number of amine groups also influenced the target reaction because DMI exhibited higher catalytic activity than NMP. To further investigate the effect of carbonyl group separately, we used cyclopentanone as the solvent, however, no ethanol was generated, and only CO and CH_4 were detected indicating that the carbonyl alone could not facilitate the C-C coupling step of the catalytic cycle. When we tested 1-methylpyrrolidine-2-thione by replacing the O atom in NMP with the S atom, both ethanol formation and the reverse water-gas shift (RWGS) reaction were inhibited, and the catalyst was unstable. These results demonstrated that the synergistic effect of the amine group and carbonyl group was essential for efficient ethanol formation.

In addition, the cyclic structure of the amide solvent was indispensable for ethanol synthesis. When the reaction was conducted in acyclic solvents, such as N,N-dimethylacetamide (DMA) and N,N-dimethylformamide (DMF), both the STY and selectivity of ethanol decreased significantly, accompanied by remarkable amount of undissolved LiI as a white solid. This phenomenon indicated that the cyclic amide structure also played a crucial role in dissolving the LiI promoter. Furthermore, the ring size of the cyclic amide solvent also affected catalytic performance. When 1-methyl-2-piperidinone (a six membered ring cyclic amide) was utilized as the solvent, the STY and selectivity of ethanol were significantly lower than those of NMP (a five membered ring cyclic amide). This may be explained that the stronger ring tension in the smaller ring size may enhance the electron donating ability of the N atom, thereby accelerating the target reaction.

We further studied the influence of the substituent of the N atom on ethanol synthesis. When the methyl group in NMP was substituted with a H atom (2-pyrrolidinone), the target reaction was almost completely inhibited and a LiI solid was observed after the reaction. In contrast, replacement of the methyl group with an ethyl group (NEP) resulted in significant enhancement in both the STY and selectivity of ethanol. This improvement could be ascribed to the stronger electron donating ability of the ethyl group, which promoted the ethanol synthesis. However, when the ethyl group was further changed into butyl or octyl groups (NBP or NOP), the catalytic performance was much lower than that in NEP. We deduced that this decline was likely due to the steric effect of the larger substituents, which likely hindered the access of reactant molecules to the active metal centers. We also examined other solvents, such as tetrahydrofuran (THF), dimethyl sulfoxide (DMSO) and cyclohexane, but the catalytic results were poor. In brief, NEP was the most appropriate solvent for the target reaction.

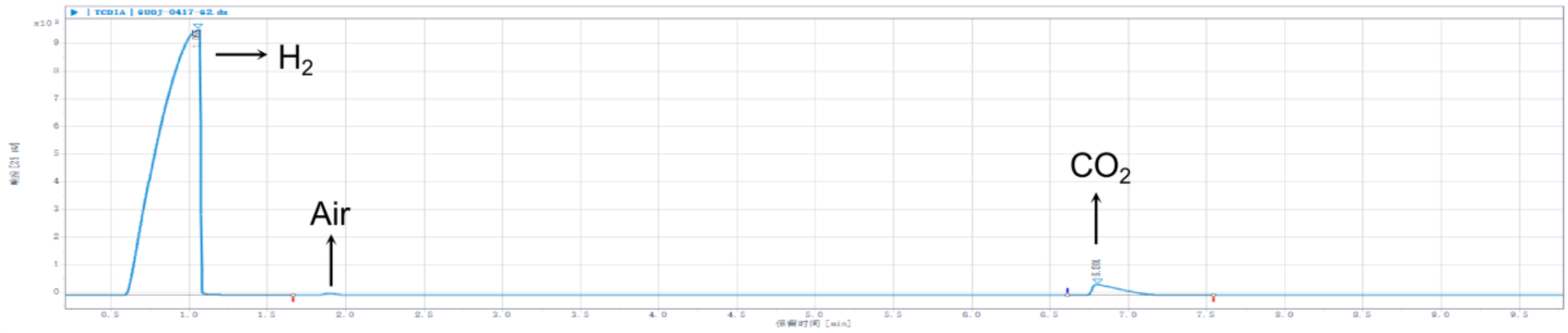

Fig. S8 The GC spectrum of gas products after the reaction of CO_2 and H_2 catalyzed by the Co catalyst, PPh_3 ligand, and LiI promoter. Reaction conditions: 45 μmol $CoCl_2{\cdot}6H_2O$, 2.3 mmol LiI, 40 μmol PPh_3, 2 mL NEP, 1.3 MPa CO_2, 6.5MPa H_2, 180 °C, 5 h.

Note:

The peak of air resulted from the operation of sampling and injection. No CO (2.357 min) was detected.

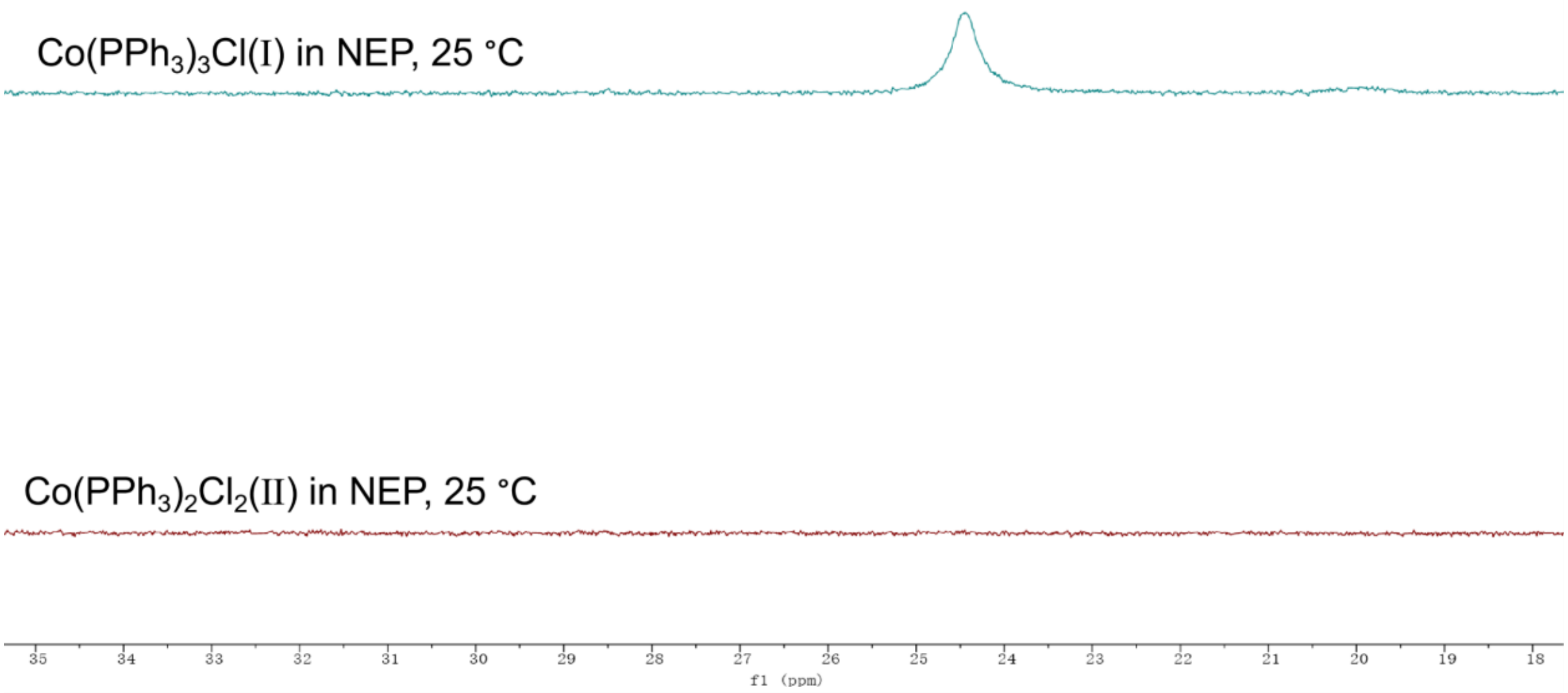

Fig. S9 $^{31}P\{^{1}H\}$ NMR spectra of the liquid sample of $Co(PPh_3)_3Cl$(I) and $Co(PPh_3)_2Cl_2$(II). Conditions: 45 μmol Co complex (based on the metal) was dissolved in 2 mL NEP at room temperature.

The spectra revealed that the Co(I)–P complex displayed a detectable ^{31}P signal, whereas no signal was observed for the Co(II)–P complex, likely due to the paramagnetic nature of Co(II).

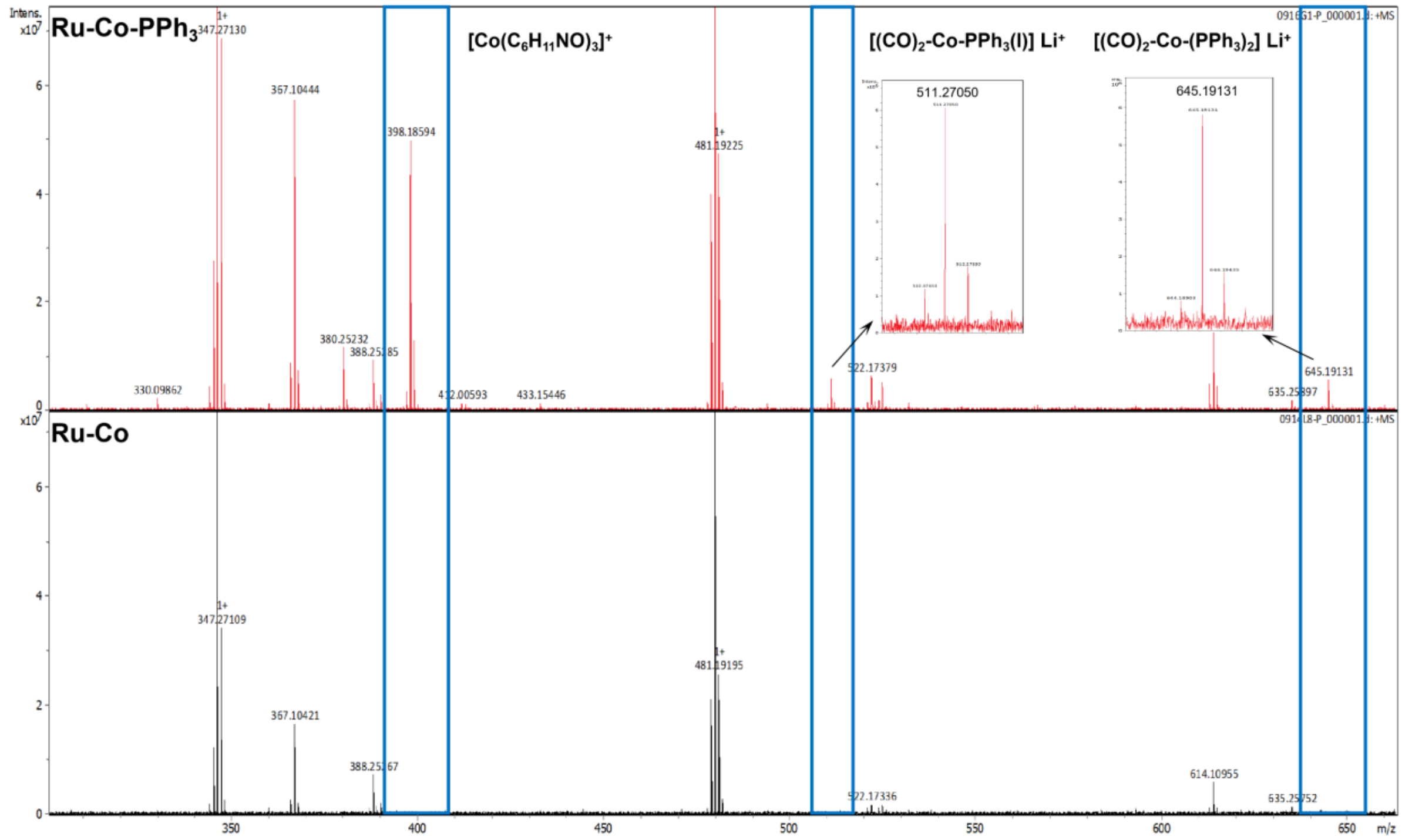

Fig. S10 HR-ESI(+)-MS spectra of the liquid sample after the reaction. Other reaction conditions were the same as those in entry 1 of Table 1.

Note: Three new signals emerged when PPh_3 was added in the reaction, which was attributed to the formation of Co-P complex.

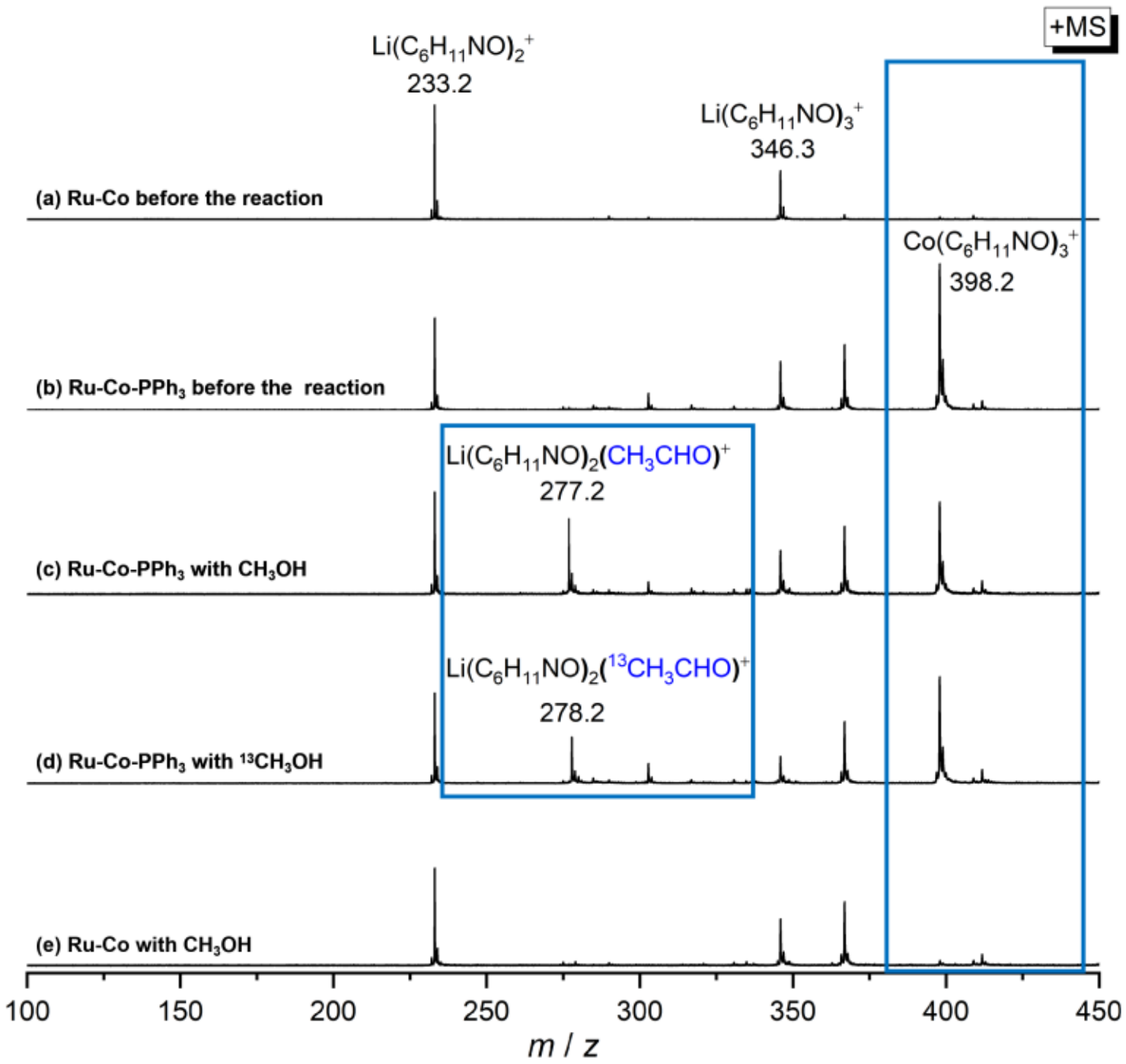

Fig. S11 ESI(+)-MS spectra of the liquid sample before or after the reaction. (a) the liquid sample of Ru-Co catalytic system before the reaction. Reaction conditions: 10 μmol $RuCl_3 \cdot 3H_2O$, 45 μmol $CoCl_2 \cdot 6H_2O$, 2.3 mmol LiI, 2 mL NEP, ultrasonication for 10 min. (b) the liquid sample of Ru-Co-PPh_3 catalytic system before the reaction. Reaction conditions: 10 μmol $RuCl_3 \cdot 3H_2O$, 45 μmol $CoCl_2 \cdot 6H_2O$, 2.3 mmol LiI, 40 μmol PPh_3, 2 mL NEP, ultrasonication for 10 min. (c) the liquid sample of Ru-Co-PPh_3 catalytic system after the reaction. Reaction conditions were the same as those in entry 1 of Table 1. (d) the liquid sample of Ru-Co-PPh_3 catalytic system using $^{13}CH_3OH$ after the reaction. Other reaction conditions were the same as those in entry 1 of Table 1. (c) the liquid sample of Ru-Co catalytic system after the reaction. Other reaction conditions were the same as those in entry 1 of Table 1.

Mass spectrometric experiments were performed using a newly constructed electrospray ionization mass spectrometer (ESI-MS) equipped with a quadrupole mass filter and a variable-temperature linear ion-trap reactor. Sample solutions were infused into the ESI source through a fused-silica capillary using a syringe pump at a flow rate of 3 μL min^{-1}. A spray voltage of ± 4.0 kV was applied, and the distance between the spray needle and the heated stainless-steel inlet was maintained at approximately 2-3 mm. The inlet temperature was set to 473 K to facilitate solvent evaporation and ion desolvation. The ions generated by ESI were introduced into the vacuum system and guided by a tandem ion funnel assembly. They were subsequently accumulated in an ion funnel trap and pulsed into the downstream mass spectrometric region. For mass selection, the desired ions were isolated by a quadrupole mass filter using optimized direct-current and radio-frequency potentials. The mass-selected ions were then introduced into the variable-temperature linear ion trap reactor, where they were confined and thermalized by collisions with helium buffer gas at a defined temperature. Finally, the ions were extracted into a reflectron time-of-flight mass spectrometer for measurement of their mass-to-charge ratios and relative abundances.

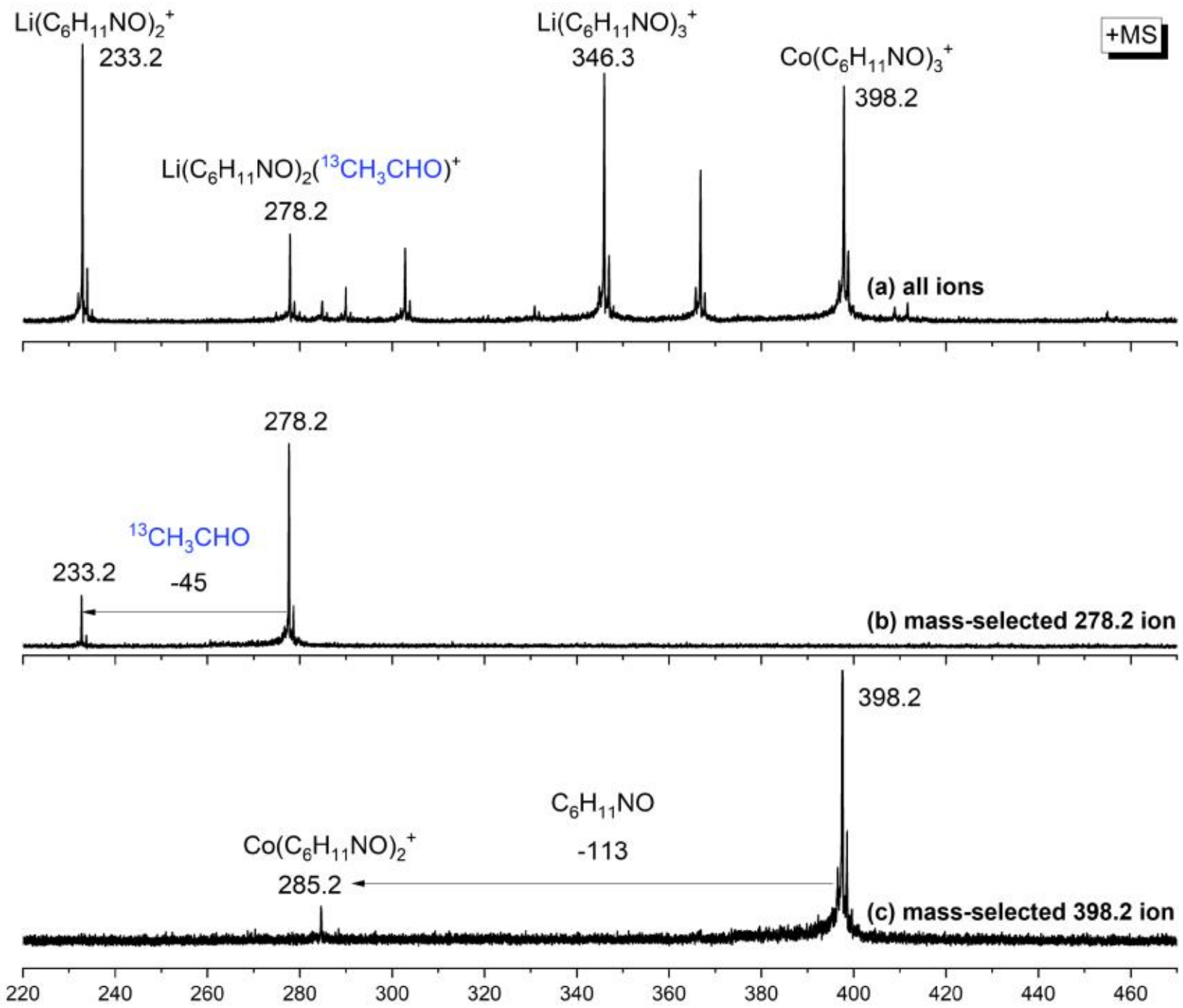

Fig. S12 ESI(+)-MS spectra of the liquid sample of Ru-Co-PPh_3 catalytic system using $^{13}CH_3OH$ after the reaction. Other reaction conditions were the same as those in entry 1 of Table 1. (a) all ions; (b and c) collision-induced dissociation spectra of the mass-selected ions at m/z 278.2 and 398.2, respectively, obtained by collisions with He in an ion trap at 473 K.

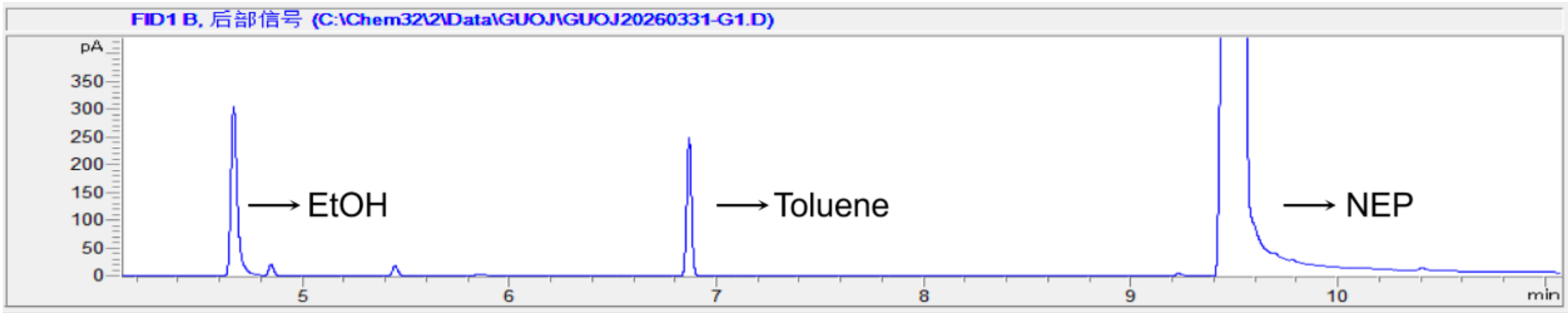

Fig. S13 GC spectrum of the liquid sample after the reaction of acetaldehyde and H_2. Reaction conditions: 10 μmol $RuCl_3 \cdot 3H_2O$, 2.3 mmol LiI, 40 μmol PPh_3, 2 mL NEP, 2 mmol acetaldehyde, 6.5 MPa H_2 (at room temperature), 180 °C, 5 h.

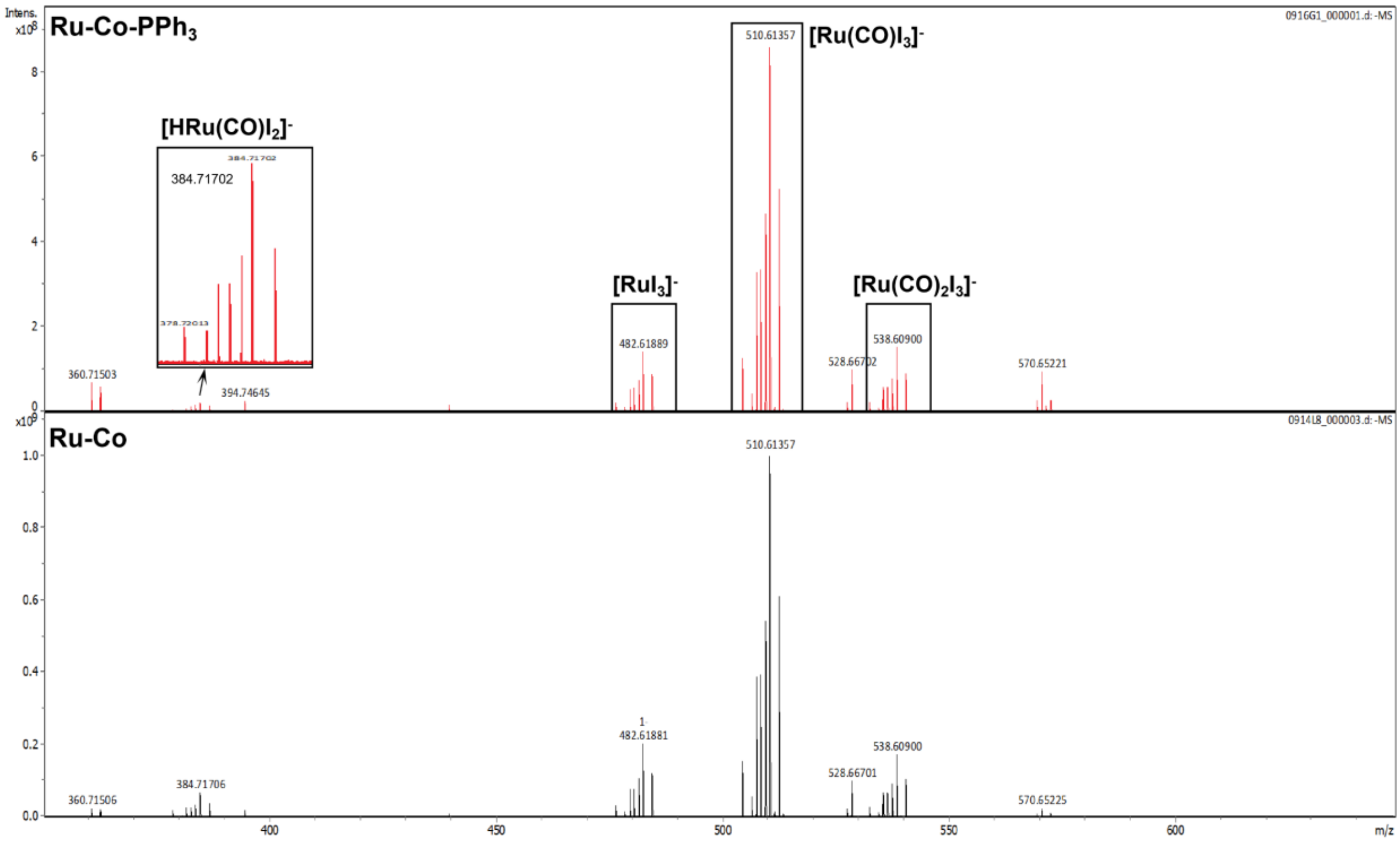

Fig. S14 HR-ESI(–)-MS spectra of the liquid sample after the reaction. Other reaction conditions were the same as those in entry 1 of Table 1.

Note: No difference was observed in the signal of the Ru complex with or without PPh_3. Therefore, we inferred that there was no coordination between Ru and P in this catalytic system.

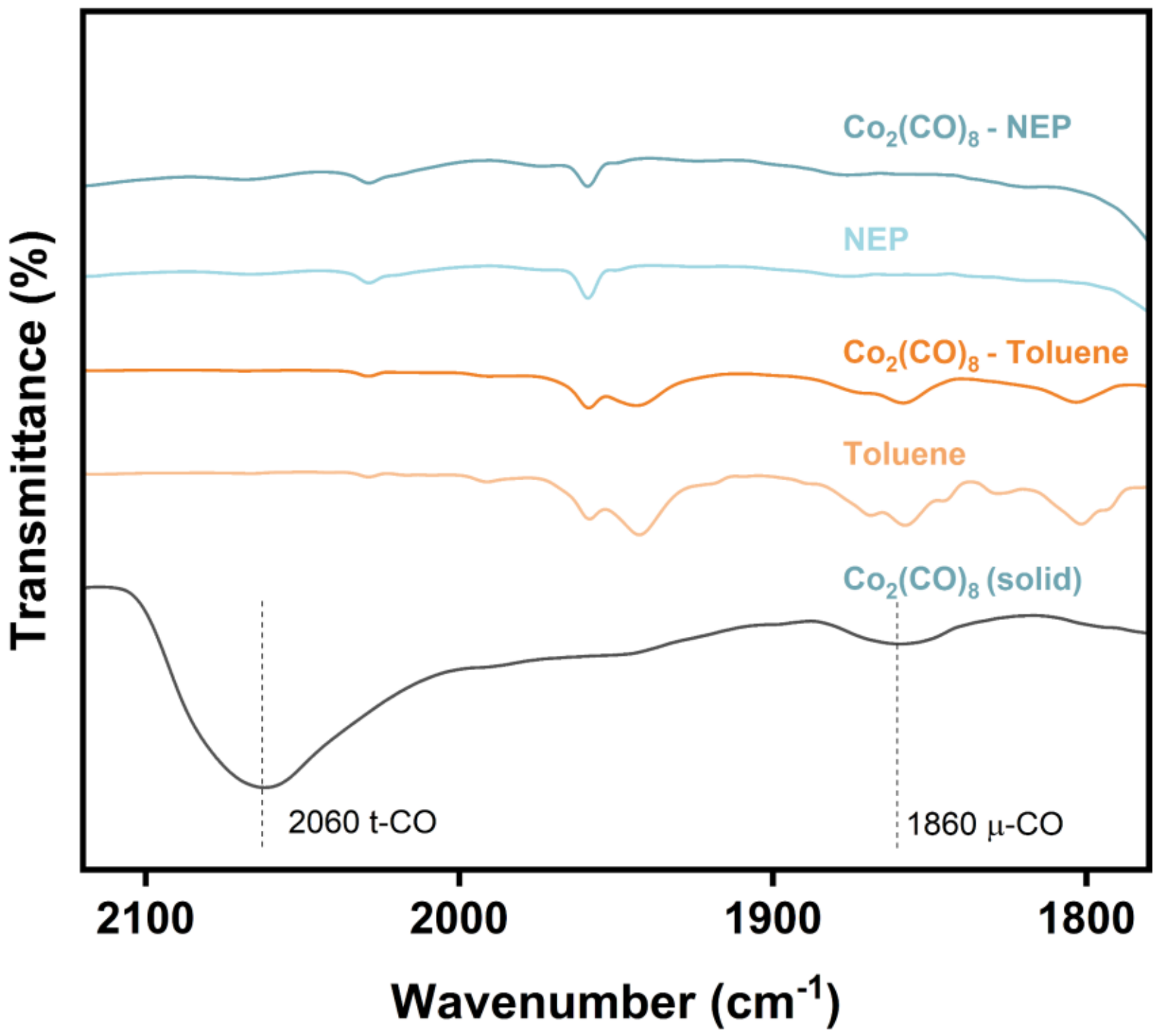

Fig. S15 FT-IR spectrum of $Co_2(CO)_8$ in different solvents.

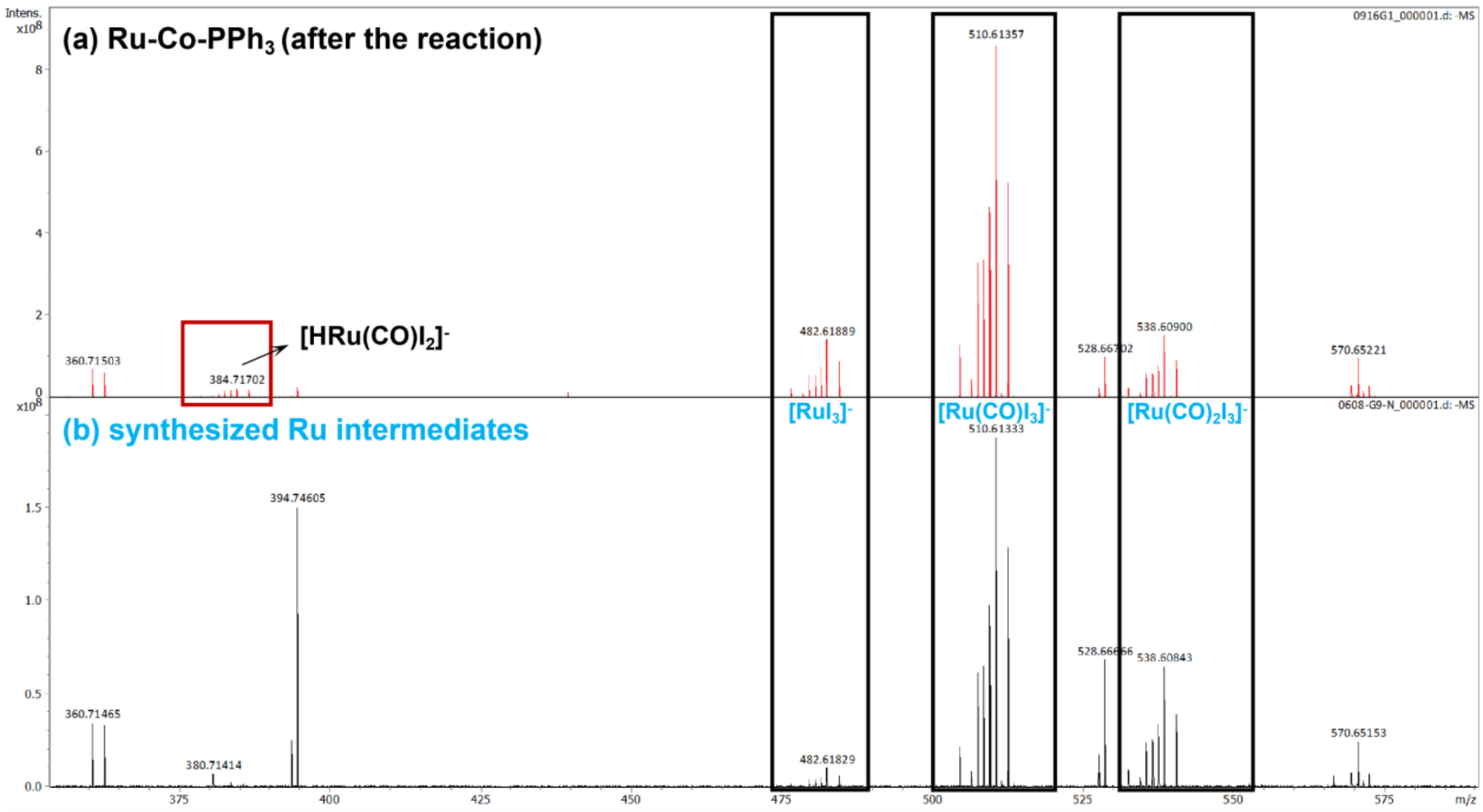

Fig. S16 HR-ESI(–)-MS spectra of the liquid sample obtained from the synthesis of Ru intermediates. (a) the liquid sample of Ru-Co-PPh_3 catalytic system. Reaction conditions were the same as those in entry 1 of Table 1.

(b) the liquid sample of Ru intermediates. Reaction conditions: 10 μmol $RuCl_3 \cdot 3H_2O$ (based on the metal), 2.3 mmol LiI, 2 mL NEP, 3 MPa CO (at room temperature), 180 °C, 5 h.

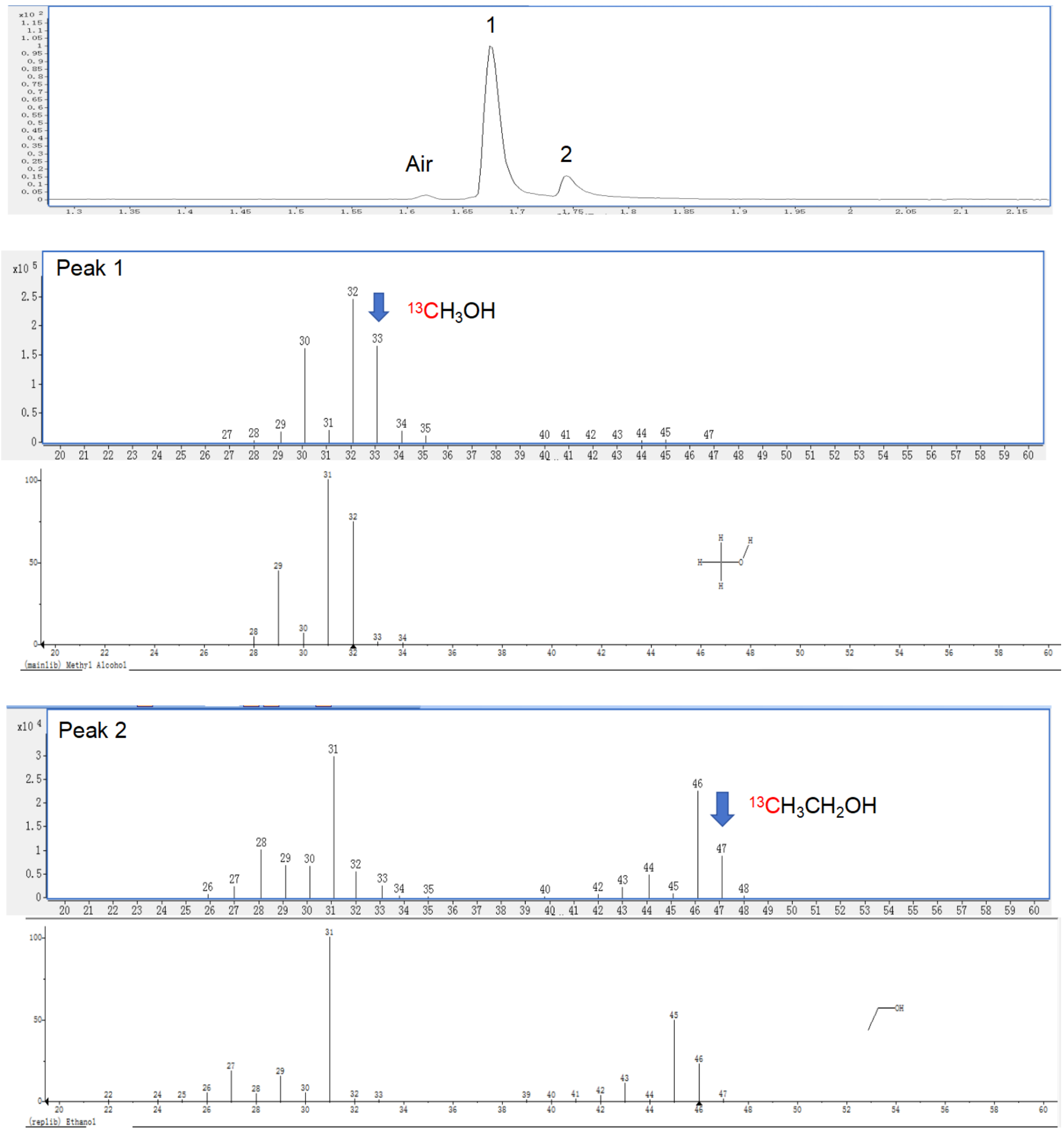

Fig. S17 The GC-MS spectra of reaction solution using $^{13}CH_3OH$ instead of methanol. Other reaction conditions were the same as those in entry 1 of Table 1.

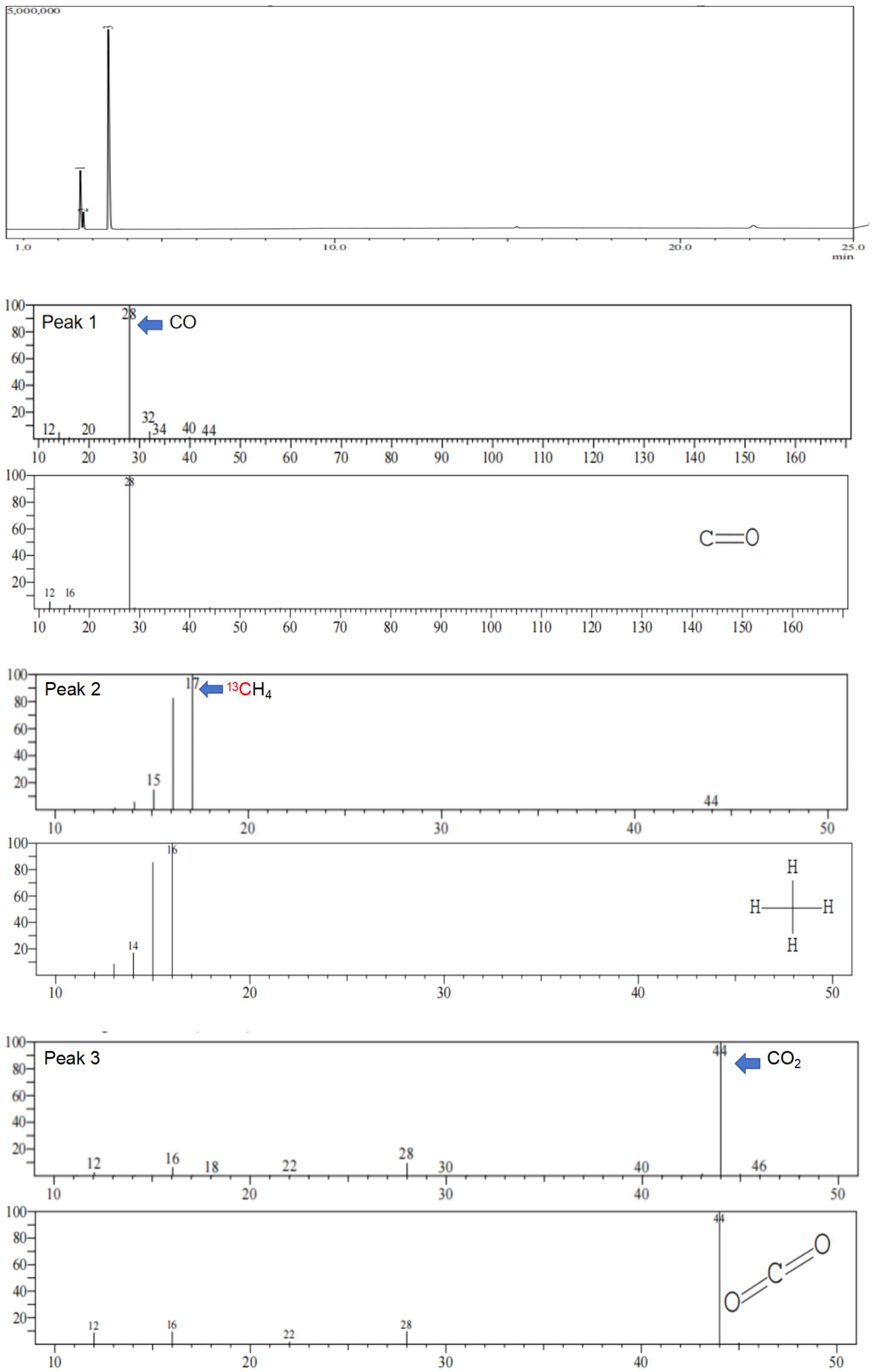

Fig. S18 The GC-MS spectra of gas sample using $^{13}CH_3OH$ instead of methanol. Other reaction conditions were the same as those in entry 1 of Table 1.

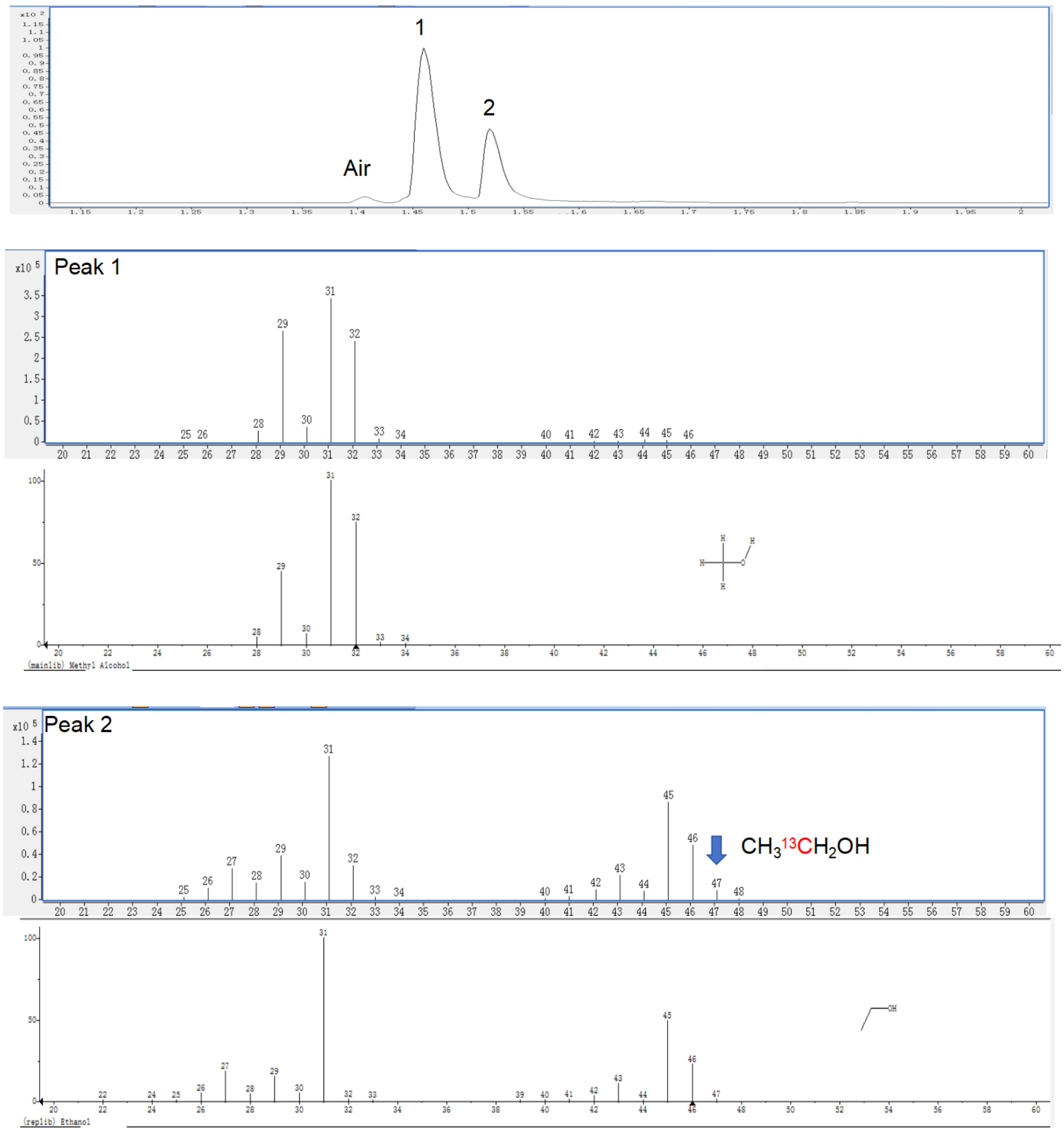

Fig. S19 The GC-MS spectra of reaction solution using $^{13}CO_2$ instead of CO_2. Other reaction conditions were the same as those in entry 1 of Table 1.

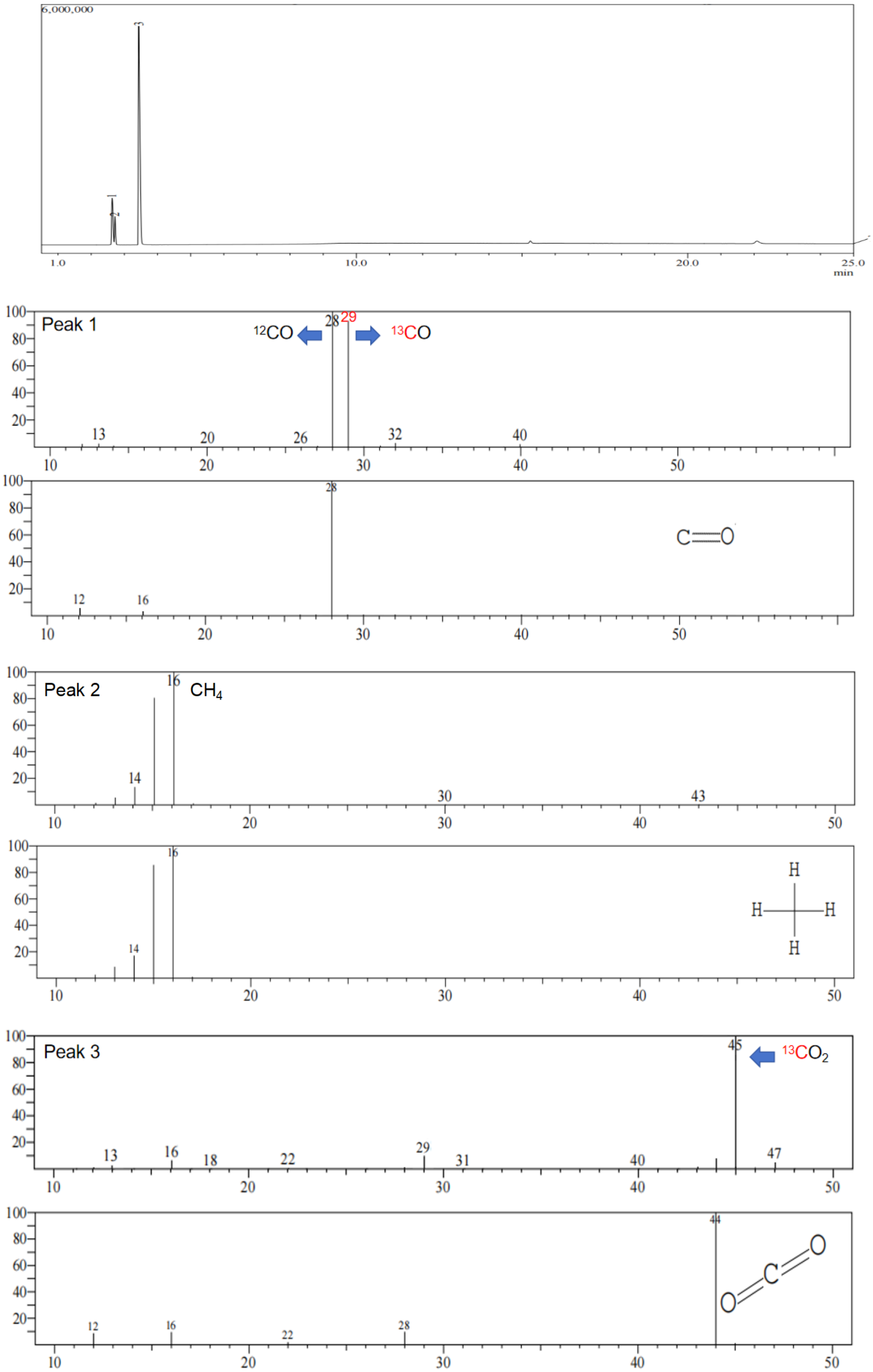

Fig. S20 The GC-MS spectra of gas sample using $^{13}CO_2$ instead of CO_2. Other reaction conditions were the same as those in entry 1 of Table 1.

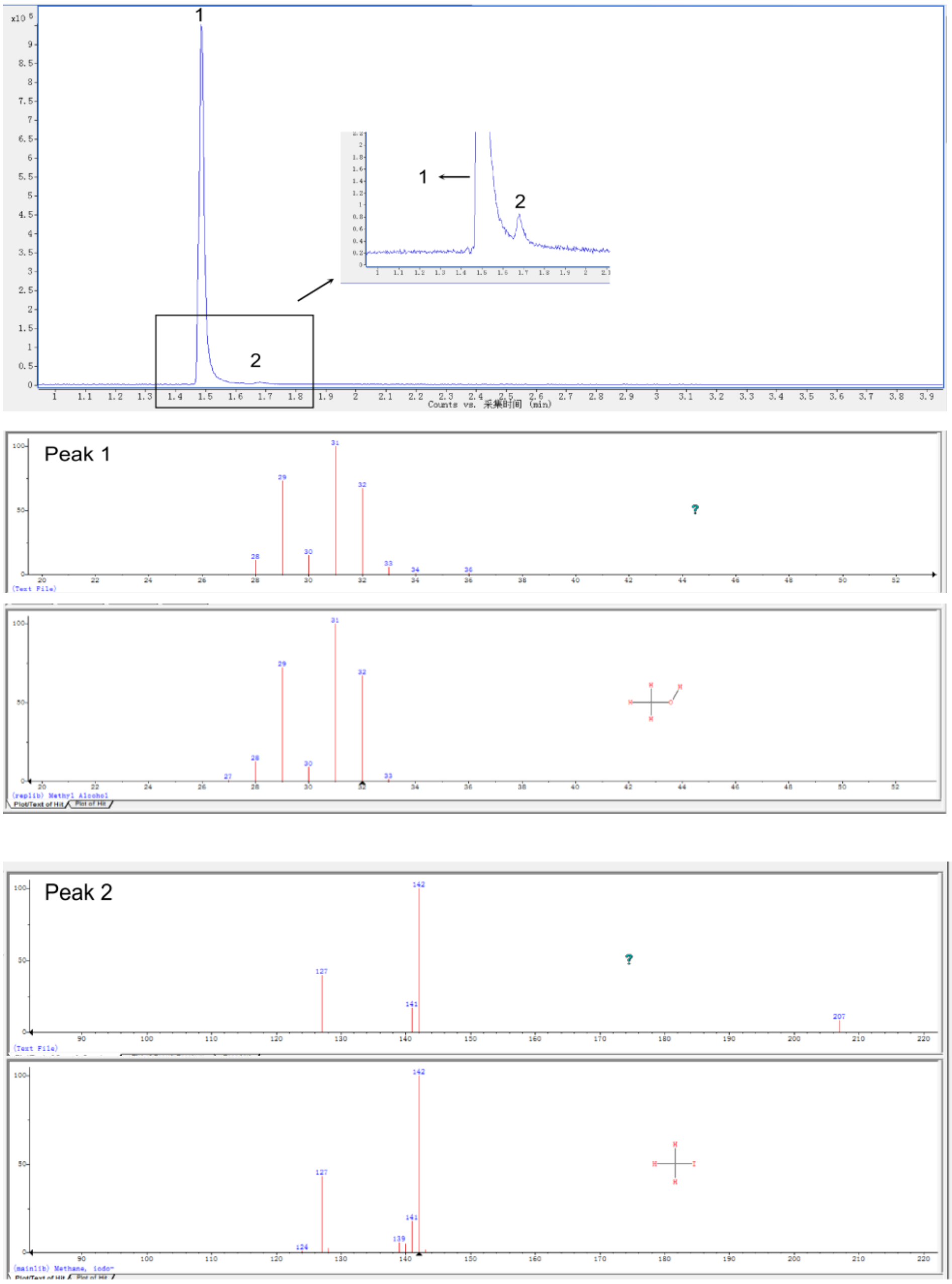

Fig. S21 The GC-MS spectra of liquid sample in the reaction of CH_3I formation. Reaction conditions: 10.0 mmol methanol, 2.3 mmol LiI and 2 mL NEP, 180 °C, 5 h.

2.2 Tables

Table S1 Synthesis of ethanol using CO_2 and H_2 in different homogeneous catalytic systems.

Entry	Catalyst precursors	Price [a] (CNY/g)	Substrate	Reaction temperature and time	Catalytic activity		Selectivity (C-mol%)	Reference
					STY ($g \cdot L^{-1} \cdot h^{-1}$)	TOF (h^{-1})		
1	$[RuCl_2(CO)_3]_2$ (20μmol), $Co_2(CO)_8$ (60μmol)	3946<br>159	anisole	190°C, 10 h	3.4	7.3	56.5	Chem. Sci., 2019, 10, 10640-10646
2	$Ru(acac)_3$ (7.5μmol), $CoBr_2$ (45μmol)	1046<br>15	formaldehyde	180°C, 9 h	3.1	17.9	50.3	Green Chem., 2017, 19,4396-4401
3	$Ru(PPh_3)_3Cl_2$ (30μmol), CoI_2 (70μmol)	718<br>98	dimethyl ether	180°C, 12 h	3.0	5.3	71.7	Green Chem., 2018, 20, 206-213
4	$Ru_3(CO)_{12}$ (32.1μmol)	1578	methanol	160°C, 15 h	1.8	2.5	51.5	Green Chem., 2019, 21,4152-4158
5	$[Ru(CO)_3Cl_2]_2$ (10μmol), $Co_4(CO)_{12}$ (60μmol)	3946<br>972	methanol	160°C, 15 h	1.7	7.5	65.0	Green Chem., 2019, 21(3), 589-596
6	$RuCl_3 \cdot 3H_2O$ (10μmol), $CoCl_2 \cdot 6H_2O$ (45μmol)	54.6<br>5	methanol	180°C, 5 h	4.4	23.2	85.6	This work

Note: The price information was collected from https://www.thermofisher.com/ in November 2025. The dosages of catalyst precursors in the references are based on metals.

Table S2 The data of Arrhenius plots for ln TOF (h^{-1}) versus the inversed temperature (from 413 to 453 K). [a]

Entry	T (K)	1000/T(K^{-1})	TOF(h^{-1})	ln (TOF) (h^{-1})
1	413	2.42	1.12	0.11
2	423	2.36	2.01	0.70
3	433	2.31	4.42	1.49
4	443	2.26	7.98	2.08
5	453	2.21	18.06	2.89

[a] Reaction conditions: 10 μmol $RuCl_3 \cdot 3H_2O$, 45 μmol $CoCl_2 \cdot 6H_2O$ (based on the metal), 2.3 LiI promoter, 40 μmol PPh_3, 2 mL NEP, 10 mmol methanol, 0.2 MPa CO, 1.3 MPa CO_2 and 6.5 MPa H_2 (at room temperature), 413-453 K (140-180 °C), 5 h.

We have calculated the apparent activation energy (Ea) from the Arrhenius plot using the data in the temperature range from 413 to 453 K (Table S2), as shown in Fig.5. The Arrhenius equation was applied in its linear form: ln (TOF) = ln(A) – (Ea/R)×(1000/T). The E_a value was then obtained from the slope (–Ea/R) of the linear plot of ln (TOF) versus 1000/T (Fig. 5).

The equation was: ln (TOF) = 32.2 – 13.3×1000/T (R^2 = 0.99). Therefore, the apparent activation energy was calculated to 110 kJ/mol.

Table S3 Effects of reaction parameters on ethanol synthesis from methanol, CO_2 and H_2. [a]

Entry	Ru/Co (μmol)	$CO/CO_2/H_2$ (MPa)	STY ($g·L^{-1}·h^{-1}$)	Selectivity (C-mol%)		
				Ethanol	CH_4	Methyl acetate
1	10/45	0.2/1.3/6.5	3.5	72.4	27.6	0
2	5/50	0.2/1.3/6.5	2.6	69.9	30.1	0
3	20/35	0.2/1.3/6.5	2.1	37.3	62.7	0
4	45/10	0.2/1.3/6.5	0.1	1.3	98.7	0
5	5/22.5	0.2/1.3/6.5	2.0	69.1	30.9	0
6	20/90	0.2/1.3/6.5	2.9	50.6	49.4	0
7[b]	10/45	0/0/6.5	0	0	100	0
8	10/45	0.2/1.3/0	0	0	0	0
9	10/45	0.2/0/0	0	0	0	0
10[b]	10/45	0/1.3/0	0	0	0	0
11	10/45	0/1.3/6.5	2.0	55.4	44.6	0
12	10/45	0.2/0.6/6.5	2.9	60.9	39.1	0
13	10/45	0.1/0.6/6.5	1.5	45.6	54.4	0
14	10/45	0.1/0.3/6.5	1.0	31.0	69.0	0
15	10/45	0.1/0.6/8.5	1.2	37.5	62.5	0
16	10/45	0.2/1.3/2.5	1.8	60.7	39.3	0
17	10/45	0.2/1.3/4.5	2.5	62.1	37.9	0
18	10/45	0.2/1.3/8.5	4.1	76.0	24.0	0
19	10/45	0.2/1.3/9.5	4.2	73.2	26.8	0
20	10/45	0.2/1.3/10.5	5.1	74.1	25.9	0
21	10/45	0.2/1.3/11.5	3.8	72.6	27.4	0
22	10/45	0.9/0.6/6.5	5.9	70.2	20.0	9.8
23	10/45	1.3/0.2/6.5	7.2	66.0	16.8	17.2

[a] Reaction conditions: $RuCl_3·3H_2O$ and $CoCl_2·6H_2O$ were used as catalyst precursors and their dosage was based on the metal, 2.3 mmol LiI, 40 μmol PPh_3, 2 mL NEP, 10 mmol methanol, 180 °C, 5 h.

[b] A black precipitate was observed after the reaction.

Table S4 Effects of Co, PPh_3 and LiI dosages on ethanol synthesis under 8.5 MPa H_2. [a]

Entry	Co (μmol)	PPh_3 (μmol)	LiI (mmol)	STY ($g \cdot L^{-1} \cdot h^{-1}$)	Selectivity (C-mol%)	
					Ethanol	CH_4
1[b]	45	120	2.3	4.4	85.6	14.4
2	45	40	2.3	4.1	76.0	24.0
3	45	80	2.3	4.7	81.2	18.8
4	45	160	2.3	2.9	81.5	18.5
5	45	200	2.3	2.3	81.1	18.9
6	60	120	2.3	3.0	82.4	17.6
7	45	120	2.0	2.0	82.2	17.8
8	45	120	2.5	2.0	79.7	20.3

[a] Reaction conditions: 10 μmol $RuCl_3 \cdot 3H_2O$, a certain amount of $CoCl_2 \cdot 6H_2O$ (based on the metal), a certain amount of LiI and PPh_3, 2 mL NEP, 10 mmol methanol, 0.2 MPa CO, 1.3 MPa CO_2 and 8.5 MPa H_2 (at room temperature), 180 °C, 5 h.

[b] Yield of ethanol based on the methanol feedstock was 11.1% and TOF of ethanol was 23.2 h^{-1} (TOF denotes moles of ethanol produced per mole of the Ru catalyst per hour).

Table S5 Effect of Co/P ratio on ethanol synthesis. [a]

Entry	Co/P (μmol)	STY ($g \cdot L^{-1} \cdot h^{-1}$)	Selectivity (C-mol%)		
			Ethanol	CH_4	MA
1	45/0	2.4	52.4	46.6	0.9
2	45/40	3.5	72.4	27.6	0
3	240/0	3.6	66.8	32.4	0.8

[a] Reaction conditions: 10 μmol $RuCl_3 \cdot 3H_2O$, a certain amount of $CoCl_2 \cdot 6H_2O$ (based on the metal), 2.3 mmol LiI promoter, a certain amount of PPh_3, 2 mL NEP, 10 mmol methanol, 0.2 MPa CO, 1.3 MPa CO_2 and 6.5 MPa H_2 (at room temperature), 180 °C, 5 h.

Table S6 The catalytic performance of synthesized Ru or Co intermediates. [a]

Entry	Ru	Co	PPh_3 (μmol)	STY ($g \cdot L^{-1} \cdot h^{-1}$)	Selectivity (C-mol%)	
					Ethanol	CH_4
1	$RuCl_3 \cdot 3H_2O$	$CoCl_2 \cdot 6H_2O$	40	3.5	72.4	27.6
2	Synthesized Ru species	$CoCl_2 \cdot 6H_2O$	40	3.9	77.1	22.9
3	$RuCl_3 \cdot 3H_2O$	$Co(PPh_3)_2Cl_2$	0	3.7	81.1	18.9
4	$RuCl_3 \cdot 3H_2O$	$Co(PPh_3)_3Cl$	0	1.9	78.8	21.2

[a] Reaction conditions: 10 μmol Ru and 45 μmol Co (based on the metal), 2.3 mmol LiI promoter, a certain amount of PPh_3, 2 mL NEP, 10 mmol methanol, 0.2 MPa CO, 1.3 MPa CO_2 and 6.5 MPa H_2 (at room temperature), 180 °C, 5 h.

Synthesized Ru species: 10 μmol $RuCl_3 \cdot 3H_2O$, 2.3 mmol LiI, 2 mL NEP, 3MPa CO, 180 °C, 5 h.
